# Numerical approach for unstructured quantum key distribution

**DOI:** 10.1038/ncomms11712

**Published:** 2016-05-20

**Authors:** Patrick J. Coles, Eric M. Metodiev, Norbert Lütkenhaus

**Affiliations:** 1 Department of Physics and Astronomy, Institute for Quantum Computing, University of Waterloo, Waterloo, Ontario, Canada N2L3G1

## Abstract

Quantum key distribution (QKD) allows for communication with security guaranteed by quantum theory. The main theoretical problem in QKD is to calculate the secret key rate for a given protocol. Analytical formulas are known for protocols with symmetries, since symmetry simplifies the analysis. However, experimental imperfections break symmetries, hence the effect of imperfections on key rates is difficult to estimate. Furthermore, it is an interesting question whether (intentionally) asymmetric protocols could outperform symmetric ones. Here we develop a robust numerical approach for calculating the key rate for arbitrary discrete-variable QKD protocols. Ultimately this will allow researchers to study ‘unstructured' protocols, that is, those that lack symmetry. Our approach relies on transforming the key rate calculation to the dual optimization problem, which markedly reduces the number of parameters and hence the calculation time. We illustrate our method by investigating some unstructured protocols for which the key rate was previously unknown.

## Abstract (web summary)

Calculating the secret key rate for a given quantum key distribution protocol is challenging. Here the authors develop a numerical approach for calculating the key rate for arbitrary discrete-variable QKD protocols, which could lead to automated security analysis of realistic systems.

## Graphical abstract

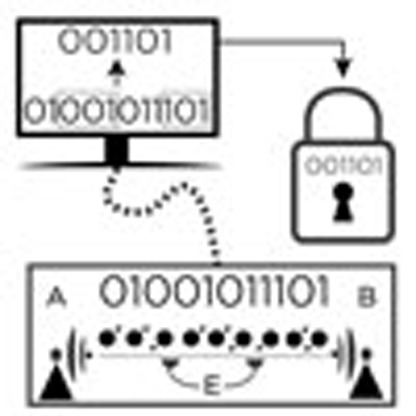

Quantum key distribution (QKD) will play an important role in quantum-safe cryptography, that is, cryptography that addresses the emerging threat of quantum computers^1^. Since its original proposal^2^,^3^, QKD has developed significantly over the past three decades^4^,^5^, both in theory and implementation. Indeed, QKD is now a commercial technology, with the prospect of global QKD networks on the horizon^6^,^7^.

The main theoretical problem in QKD is to calculate how much secret key can be distributed by a given protocol. A crucial practical issue is that the QKD protocols that are easiest to implement with existing optical technology do not necessarily coincide with the protocols that are easiest to analyse theoretically^4^. Currently, calculating the secret key output of a protocol is typically extremely technical, and hence only performed by skilled experts. Furthermore, each new protocol idea requires a new calculation, tailored to that protocol. Ultimately, the technical nature of these calculations combined with the lack of universal tools limits the pace at which new QKD protocols can be discovered and analysed. Here we address this problem by developing a robust, user-friendly framework for calculating the secret key output, with the hope of bringing such calculations ‘to the masses'.

The secret key output is typically quantified by the key rate, which refers to the number of bits of secret key established divided by the number of distributed quantum systems. Operationally, this corresponds to the question of how much privacy amplification Alice and Bob must apply to transform their raw key into the final secure key. Analytical simplifications of the key rate calculation can be made for some special protocols that have a high degree of symmetry^8^. Examples of such symmetric protocols, where the signal states have a group-theoretic structure, include the BB84 (ref. 3) and six-state protocols^9^. Indeed the key rate is known for these protocols. However, in practice, lack of symmetry is the rule rather than the exception. That is, even if experimentalists try to implement a symmetric protocol, experimental imperfections tend to break symmetries^10^. Furthermore, it is sometimes desirable due to optical hardware issues to implement asymmetric protocols, for example, as in ref. 11.

We refer to general QKD protocols involving signal states or measurement choices that lack symmetry as ‘unstructured' protocols. Some recent work has made progress in bounding the key rate for special kinds of unstructured protocols, such as four-state protocols in refs 12, 13 and qubit protocols in ref. 14. Still, there is no general method for computing tight bounds on the key rate for arbitrary unstructured protocols. Yet, these are the protocols that are most relevant to experimental implementations.

This motivates our present work, in which we develop an efficient, numerical approach to calculating key rates. Our ultimate aim is to develop a computer program, where Alice and Bob input a description of their protocol (for example, their signal states, measurement devices, sifting procedure and key map) and their experimental observations, and the computer outputs the key rate for their protocol. This program would allow for any protocol, including those that lack structure.

At the technical level, the key rate problem is an optimization problem, since one must minimize the well-known entropic formula for the key rate^15^, over all states *ρ*_*AB*_ that satisfy Alice's and Bob's experimental data. The main challenge here is that this optimization problem is unreliable and inefficient. In this work, we give a novel insight that transforming to the dual problem (for example, see ref. 16) resolves these issues, hence paving the way for automated key rate calculations.

Specifically, the unreliable (or unphysical) aspect of the primal problem is that it is a minimization, hence the output will in general be an upper bound on the key rate. But one is typically more interested in reliable lower bounds, that is, physically achievable key rates. Transforming to the dual problem allows one to formulate the problem as a maximization, and hence approach the key rate from below. Therefore, every number outputted from our computer program represents an achievable asymptotic key rate, even if the computer did not reach the global maximum.

The inefficient aspect of the primal problem is that the number of parameters grows as 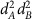 for a state *ρ*_*AB*_ with 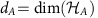 and 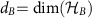. For example, if *d*_*A*_=*d*_*B*_=10, the number of parameters that one would have to optimize over is 10,000. In contrast, in the dual problem, the number of parameters is equal to the number of experimental constraints that Alice and Bob choose to impose. For example, in the generalization of the BB84 protocol to arbitrary dimensions^17^,^18^, Alice and Bob typically consider two constraints, their error rates in the two mutually-unbiased bases (MUBs). So, for this protocol, we have reduced the number of parameters to something that is constant in dimension. We, therefore, believe that our approach (of solving the dual problem) is ideally suited to efficiently calculate key rates in high dimensions.

We have written a MATLAB program to implement our key rate calculations. To illustrate the validity of our program, we show (Fig. 1) that it exactly reproduces the known theoretical dependence of the key rate on error rate, for both the BB84 and six-state protocols.

But ultimately the strength of our approach is its ability to handle unstructured protocols. We demonstrate this by investigating some unstructured protocols for which the key rates were not previously known. For example, we study a general class of protocols where Alice and Bob measure *n* MUBs, with 2≤*n*≤*d*+1, in dimension *d*. Also, we investigate the B92 protocol^19^, which involves two signal states whose inner product is arbitrary. Our key rates are higher than known analytical lower bounds^20^,^21^ for B92. Finally, we argue that our approach typically gives markedly higher key rates than those obtained from an analytical approach based on the entropic uncertainty relation^22^,^23^.

We focus on asymptotic key rates in this work. Nevertheless, the optimization problem that we solve is also at the heart of finite-key analysis, for example, see refs 24, 25. We, therefore, hope to extend our approach to the finite-key scenario in future efforts. We remark that finite-size effects generally reduce the key rate below its asymptotic value.

In what follows, we first present our main result: a reformulation of the key rate optimization problem in such a way that it is easily computable. We then outline our general framework for treating a broad range of protocols. Finally, we illustrate our approach with various examples.

## Results

### Setup of the problem

Consider a general entanglement-based QKD protocol involving finite-dimensional quantum systems *A* and *B* that are respectively received by Alice and Bob. Note that prepare-and-measure QKD protocols can be recast as entanglement-based protocols, as discussed below. For simplicity of presentation, we consider protocols where Alice's raw key is derived from a measurement on her system, possibly after some post-selection corresponding to a public announcement with a binary outcome, ‘pass' or ‘fail'. However, our approach can easily be extended to more general protocols.

Let *Z*_*A*_ (*Z*_*B*_) denote the measurement that Alice (Bob) performs on system *A* (*B*) to derive the raw key. Suppose they use one-way direct reconciliation for the classical post-processing and that their error correction is optimal (that is, leaks out the minimum number of bits), then the asymptotic key rate is given by the Devetak–Winter formula^15^:


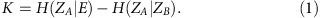


In equation (1), *H*(*X*|*Y*):=*H*(*ρ*_*XY*_)−*H*(*ρ*_*Y*_) is the conditional von Neumann entropy, with 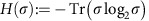, and


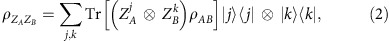



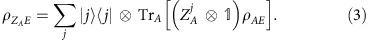


Here *ρ*_*ABE*_ is the tripartite density operator shared by Alice, Bob and Eve (and it may be the state after some post-selection procedure, see our general framework below). Also, 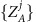 and 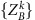 are the sets of positive operator valued measure (POVM) elements associated with Alice's and Bob's key-generating measurements. In what follows we refer to 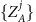 as the key-map POVM.

In the previous paragraph and in what follows, we assume that the state shared by Alice, Bob and Eve has an i.i.d. (independent, identically distributed) structure, and hence it makes sense to discuss the state *ρ*_*ABE*_ associated with a single round of quantum communication. To avoid confusion, we emphasize that our approach is ‘unstructured' in the sense of lacking structure for a given round of quantum communication, but we do impose the i.i.d. structure that relates one round to other rounds. This i.i.d. structure corresponds to Eve doing a so-called collective attack. However, the security of our derived asymptotic key rate also holds against the most general attacks (coherent attacks) if one imposes that the protocol involves a random permutation of the rounds (a symmetrization step) such that the de Finetti theorem^26^,^27^ or the post-selection technique^28^ applies.

Typically, Alice's and Bob's shared density operator *ρ*_*AB*_ is unknown to them. A standard part of QKD protocols is for Alice and Bob to gather data through local measurements, and in a procedure known as parameter estimation, they use this data to constrain the form of *ρ*_*AB*_. The measurements used for this purpose can, in general, be described by bounded Hermitian operators Γ_*i*_, with the set of such operators denoted by 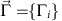.

From their data, Alice and Bob determine the average value of each of these measurements:


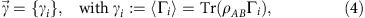


and this gives a set of experimental constraints:


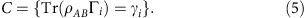


We denote the set of density operators that are consistent with these constraints as:


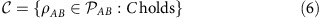


where 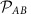 denotes the set of positive semidefinite operators on 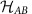, and an additional constraint 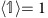 is assumed to be added to the set *C* to enforce normalization.

Because Alice and Bob typically do not perform full tomography on the state, 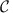 includes many density operators, and hence the term *H*(*Z*_*A*_|*E*) in equation (1) is unknown. To evaluate the key rate, Alice and Bob must consider the most pessimistic of scenarios where *H*(*Z*_*A*_|*E*) takes on its smallest possible value that is consistent with their data. This is a constrained optimization problem, given by


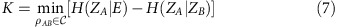


where Eve's system *E* can be assumed to purify *ρ*_*AB*_ since it gives Eve the most information. Here the number of parameters in the optimization is (*d*_*A*_*d*_*B*_)^2^, corresponding to the number of parameters in a positive semidefinite operator on 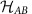. We refer to equation (7) as the primal problem.

### Main result

Our main result is a reformulation of the optimization problem in equation (7).

*Theorem 1*: The solution of the minimization problem in equation (7) is lower bounded by the following maximization problem:


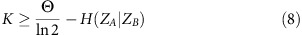


where


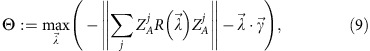


and


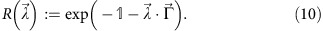


In equation (9), the optimization is over all vectors 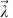
={*λ*_*i*_}, where the *λ*_*i*_ are arbitrary real numbers and the cardinality of 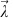 is equal to that of 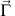. Also, 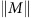 denotes the supremum norm of *M*, which is the maximum eigenvalue of *M* when *M* is positive semidefinite, as in equation (9).

The proof of Theorem 1 is given in the Methods section. It relies on the duality of convex optimization problems, as well as some entropic identities that allow us to simplify the dual problem. Note that the term *H*(*Z*_*A*_|*Z*_*B*_) in equation (8) is pulled outside of the optimization since Alice and Bob can compute it directly from their data.

The cardinalities of the sets 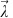 and 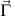 are the same. This means that the number of parameters *λ*_*i*_ that one must optimize over, to solve equation (9), is equal to the number of experimental constraints that Alice and Bob have. (More precisely this is the number of independent constraints, since one can eliminate constraints that carry redundant information.) This has the potential to be significantly less than the number of parameters in the primal problem. Indeed, we demonstrate below that equation (9) can be easily solved using MATLAB on a personal computer for a variety of interesting QKD protocols.

### Formulating constraints

For a given protocol, how does one decide which constraints to include in the set *C*? Consider the following remarks. First, adding in more constraints will never decrease the key rate obtained from our optimization. This follows since adding a new constraint gives an additional *λ*_*i*_ to maximize over, while setting this new *λ*_*i*_ to zero recovers the old problem. Second, coarse-graining constraints, that is, merging two constraints 〈Γ_*i*_〉=*γ*_*i*_ and 〈Γ_*j*_〉=*γ*_*j*_ into a single constraint 〈Γ_*i*_+Γ_*j*_〉=*γ*_*i*_+*γ*_*j*_, will never increase the key rate obtained from our optimization. This follows since merging two constraints means that two *λ*_*i*_'s are merged into a single *λ*_*i*_, thus restricting the optimization. Hence, to obtain the highest key rates, one should input all of one's refined knowledge that is available into our optimization. On the other hand, coarse graining reduces the number of constraints and thus may help to simplify the optimization problem, possibly at the cost of a reduced key rate.

One's refined knowledge is captured as follows. In a general entanglement-based protocol, Alice measures a POVM (whose elements may be non-commuting, for example, if she randomly measures one of two MUBs), which we denote as Γ_*A*_={Γ_*A*,*i*_}. Likewise Bob's corresponding POVM is Γ_*B*_={Γ_*B*,*i*_}. Hence, through public discussion, Alice and Bob obtain knowledge of expectation values of the form


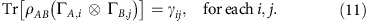


These constraints form the set *C* in equation (5). We remark that it is common in the QKD field to express correlations in terms of average error rates rather than in terms of the joint probability distribution in equation (11). This is an example of the coarse graining that we mentioned above. For simplicity of presentation, we will do this sort of coarse graining for some protocols that we investigate below, although equation (11) represents our general framework for constructing *C*.

### Framework for prepare and measure

While our approach is stated in the entanglement-based scenario, let us note how it applies to prepare-and-measure protocols. Consider a prepare-and-measure protocol involving a set of *N* signal states {|*φ*_*j*_〉}, which Alice sends with probabilities {*p*_*j*_}. It is well-known that prepare-and-measure protocols can be recast as entanglement-based protocols using the source-replacement scheme (see, for example, refs 4, 8, 29). Namely, one forms the entangled state:


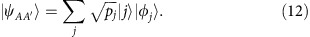


One imagines that Alice keeps system *A*, while system *A*′ is sent over an insecure quantum channel 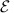 to Bob, resulting in


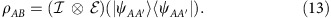


The numerical optimization approach described above can then be applied to the state *ρ*_*AB*_ in equation (13). However, in addition to the constraints obtained from Alice's and Bob's measurement results, we must add in further constraints to account for the special form of *ρ*_*AB*_. In particular, note that the partial trace over *B* gives


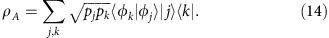


The form of *ρ*_*A*_, which is closely related to Gram matrix, depends on the inner products between the signal states, which (we assume) Alice knows. Suppose {Ω_*i*_} is a set of tomographically complete observables on system *A*, then one can add in the calculated expectation values {*ω*_*i*_} of these observables into the set of constraints. That is, add


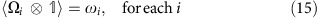


to the set *C* in equation (5). This will capture Alice's knowledge of her reduced density operator.

### Framework for decoy states

In decoy-state QKD^30^, which aims to combat photon-number splitting attacks, Alice prepares coherent states of various intensities and then randomizes their phases before sending them to Bob. Our framework can handle decoy states simply by allowing for additional signal states to be added to the set {|*φ*_*j*_〉} in equation (12). For example, to treat decoy protocols with partial phase randomization^31^, one can consider signal states that are bipartite (on the signal mode *S* and the reference mode *R*) of the form


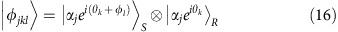


where *α*_*j*_ is the amplitude of the coherent state associated with the *j*th intensity setting, *θ*_*k*_ is the *k*th phase used in phase randomization, and *φ*_*l*_ is the phase Alice uses to encode her information (for example, for generating key). Decoy protocols with complete phase randomization are also treatable in our framework, namely, by adding in a signal state for each photon-number basis state (up to a cutoff), and treating multi-photon signals as orthogonal states (so-called ‘tagged states') since Eve can perfectly distinguish them.

### Framework for MDI QKD

A special kind of prepare-and-measure protocol is measurement-device-independent (MDI) QKD^32^. In MDI QKD, Alice prepares states {|*φ*_*j*_〉} with probabilities {*p*_*j*_} and sends them to Charlie, and Bob does the same procedure as Alice (Fig. 2). Charlie typically does a Bell-basis measurement, however the security proof does not assume this particular form of measurement. Charlie announces the outcome of his measurement, which we denote by the classical register *M*. Our framework for treating MDI QKD considers the tripartite state *ρ*_*ABM*_, where *A* and *B*, respectively, are Alice's and Bob's systems in the source-replacement scheme, playing the same role as system *A* in equation (12) (see Supplementary Note 1 for elaboration). For our numerics, we impose the constraint that the marginal *ρ*_*AB*_=*ρ*_*A*_⊗*ρ*_*B*_ is fixed (since Eve cannot access *A* and *B*), with *ρ*_*A*_ and *ρ*_*B*_ given by the form in equation (14). We enforce this constraint using the same approach as used in equation (15) to fix *ρ*_*A*_ for prepare-and-measure protocols. The only other constraints we impose are the usual correlation constraints, that is, a description of the joint probability distribution for the standard bases on *A*, *B* and *M*, of the form


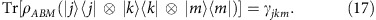


### Framework for post-selection and announcements

In general, a QKD protocol may involve post-selection. As an example, if Alice sends photons to Bob over a lossy channel, then they may post-select on rounds in which Bob detects a photon. As noted above, for simplicity we consider protocols where the post-selection involves a binary announcement, and Alice and Bob keep (discard) rounds when ‘pass' (‘fail') is announced. Let 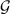 be the completely positive linear map corresponding to the post-selection. The action of 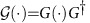 is given by a single Kraus operator *G*, corresponding to the ‘pass' announcement.

The key rate formula (1) should be applied to the post-selected state:


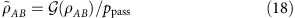


where 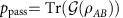 is the probability for passing the post-selection filter. We remark that since 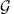 is given by a single Kraus operator, it maps pure states to pure states, and hence taking Eve's system to purify the post-selected state 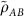 is equivalent to taking it to purify *ρ*_*AB*_. Hence applying the key rate formula to 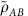 does not give Eve access to any more than she already has, and hence does not introduce any looseness into our bound. Future extension of our work to more general maps 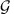 will need to carefully account for how Eve's system is affected by 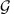, so as not to lose key rate from this proof technique.

The only issue is that Alice's and Bob's experimental constraints *C* in equation (5) are still in terms of state *ρ*_*AB*_. To solve for the key rate, one must transform these constraints into constraints on 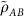. For the special case where 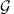 has an inverse 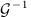 that is also completely positive, one can simply insert the identity channel 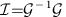 into the expression 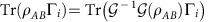. Using cyclic permutation under the trace, we transform equation (5) into a set of constraints on 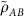,


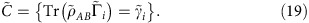


where the 
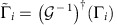
 are Hermitian operators, with 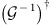 being the adjoint of 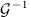, and 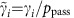. Note that *p*_pass_ is determined experimentally and hence the 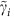 are known to Alice and Bob. More generally, we provide a method for obtaining 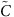 for arbitrary 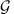, as described in Supplementary Note 2.

We remark that public announcements can be treated with a simple extension of our post-selection framework. While our framework directly applies to announcements with only two outcomes corresponding to ‘pass' or ‘fail' (as discussed above), more general announcements can be treated by adding classical registers that store the announcement outcomes. Our treatment of MDI QKD is an example of this approach (Fig. 2 and Supplementary Note 1). Additional examples that could be treated in this way are protocols with two-way classical communication^33^ such as advantage distillation.

### Outline of examples

**Figure 1:**
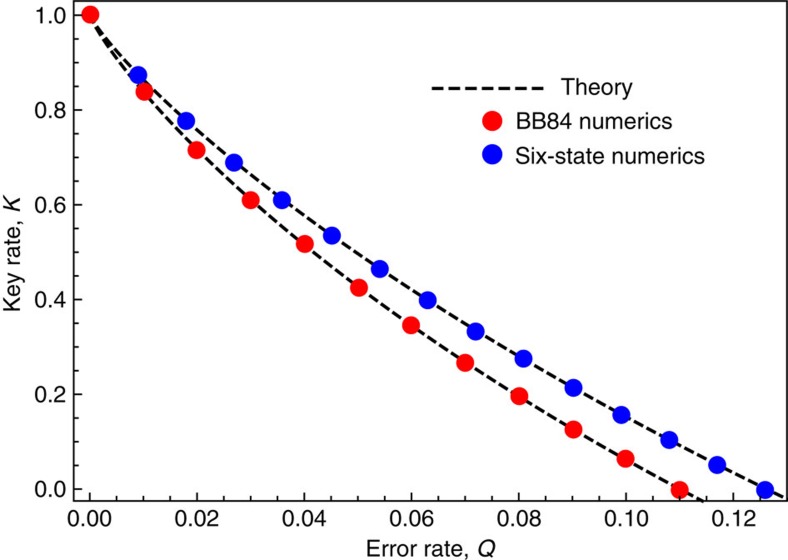
Key rate for two well-known QKD protocols. Here we compare our numerics (from Theorem 1) with the theoretical curves. The results of our numerical optimization for the BB84 and six-state protocols are respectively shown as red and blue dots. The known theoretical curves for these protocols are also shown as black dashed lines. The dots should be viewed as reliable lower bounds on the key rate, but in this case they happen to be perfectly tight, coinciding with the theoretical curves.

**Figure 2:**
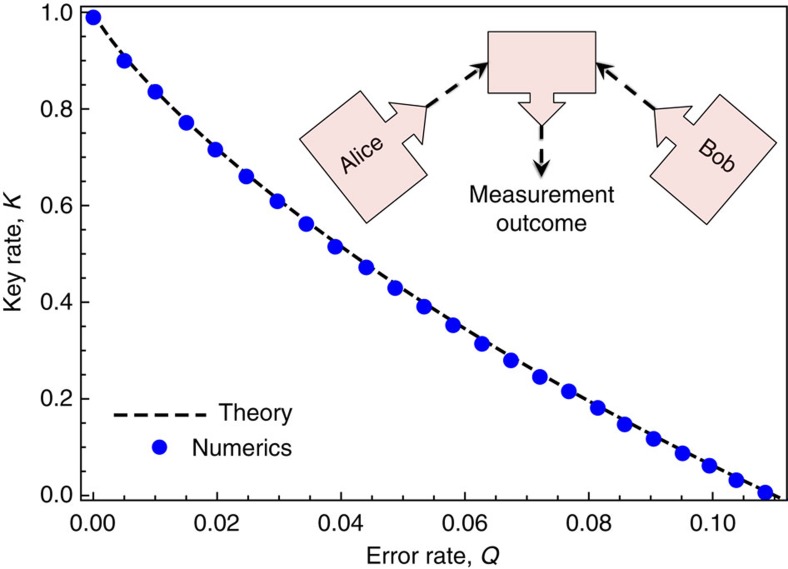
Key rate for MDI QKD with the BB84 signal states. The inset shows the basic idea of MDI QKD: Alice and Bob each prepare a signal state and send it to an untrusted node, which performs an (untrusted) Bell-basis measurement and announces the outcome. Our numerics (circular dots) essentially reproduce the known theoretical dependence of the key rate on the error rate (dashed curve), which is the same expression as that given in (20). See Supplementary Note 1 for elaboration.

**Figure 3:**
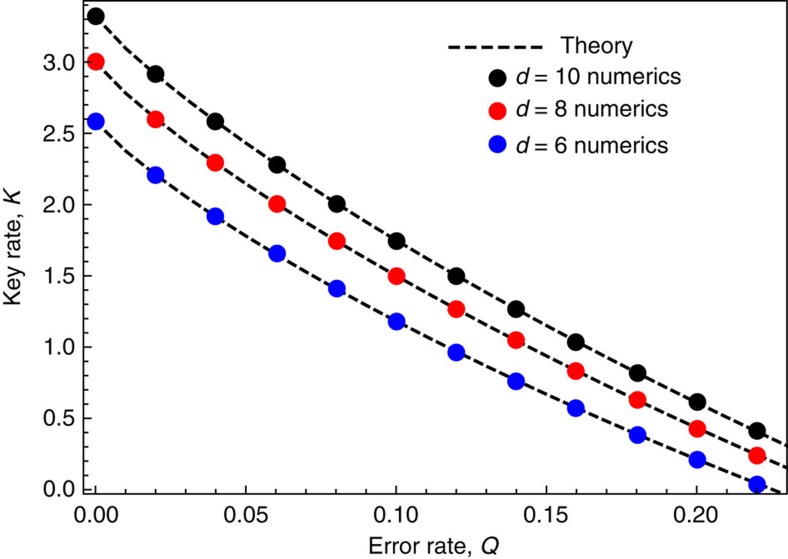
Higher dimensional analog of BB84, using two MUBs. This plot shows the theoretical key rate as solid curves, and the result of our numerical optimization as circular dots, for *d*_*A*_=*d*_*B*_=*d*, with *d*=6 (blue), *d*=8 (red), and *d*=10 (black). Again, the dots should be viewed as reliable lower bounds, but in this case they are perfectly tight.

We now illustrate our numerical approach for lower bounding the key rate by considering some well-known protocols. First, we consider the BB84 and six-state protocols (Fig. 1), MDI QKD with BB84 states (Fig. 2), and the generalized BB84 protocol involving two MUBs in any dimension (Fig. 3). In each case, the dependence of the key rate on error rate is known, and we show that our numerical approach exactly reproduces these theoretical dependences. After considering these structured protocols, we move on to using our numerical optimization for its intended purpose: studying unstructured protocols. The fact that our bound is tight for the structured protocols mentioned above gives reason to suspect that we will get strong bounds in the unstructured case. We investigate below a protocol involving *n* MUBs, a protocol involving bases with arbitrary angle between them, and the B92 protocol.

### BB84 example

Consider an entanglement-based version of the BB84 protocol^3^, where Alice and Bob each receive a qubit and measure either in the *Z*={|0〉, |1〉} or *X*={|+〉, |−〉} basis, where 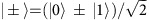. For all protocols that we discuss, we assume perfect sifting efficiency, which can be accomplished asymptotically via asymmetric basis choice^34^. Let us suppose that Alice and Bob each use their *Z* basis to generate key. For simplicity, suppose they observe that their error rates in the *Z* and *X* bases are identical and equal to *Q*, then it is known (see, for example, ref. 4) that the key rate is given by


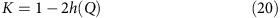


where 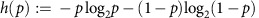 is the binary entropy.

To reproduce this result using our numerics, we write the optimization problem as follows:


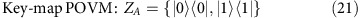



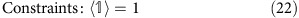



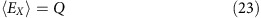



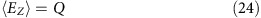


where the error operators are defined as


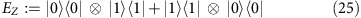



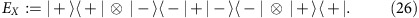


Equations (21)–(24), 65, 66, 67 highlight the fact that, as far as the optimization in equation (9) is concerned, a QKD protocol is defined by the POVM elements used for generating the key and the experimental constraints used for ‘parameter estimation' (and also the post-selection map 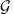, but this is trivial for the ideal BB84 protocol). Once these things are specified, the protocol is defined and the key rate is determined. Numerically solving the problem defined in equations (21)–(24) for several values of *Q* leads to the red dots in Fig. 1, which agree perfectly with the theory curve.

### Six state example

Now consider an entanglement-based version of the six-state protocol, where Alice and Bob each measure one of three MUBs (*X*, *Y* or *Z*) on their qubit. Suppose that Alice and Bob observe that their error rates in all three bases are identical, 〈*E*_*X*_〉=〈*E*_*Y*_〉=〈*E*_*Z*_〉=*Q*, where


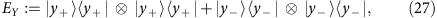


with 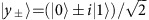. (Our definition of *E*_*Y*_ reflects the fact that the standard Bell state is correlated in *Z* and *X* but anti-correlated in *Y*.) To reproduce the known key rate^9^,^21^, we write the optimization problem as:


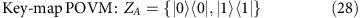



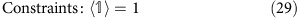



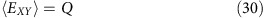



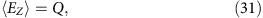


where *E*_*XY*_ :=(*E*_*X*_+*E*_*Y*_)/2 quantifies the average error for *X* and *Y*. Note that the constraint 〈*E*_*XY*_〉=*Q* is obtained by coarse graining the individual error rates. In theory, one can get a stronger bound on the key rate by splitting up this constraint into 〈*E*_*X*_〉=*Q* and 〈*E*_*Y*_〉=*Q*. However, our numerics show that this does not improve the key rate, and the constraints in equation (29)–(31) are enough to reproduce the theory curve. Indeed, numerically solving the problem in equation (28)–(31) leads to the blue dots in Fig. 1, which agree with the theory curve.

### Two MUBs in higher dimensions example

A distinct advantage of our approach of solving equation (9) instead of the primal problem equation (7) is that we can easily perform the optimization in higher dimensions, where the number of parameters in equation (7) would be quite large. To illustrate this, we consider a generalization of BB84 to arbitrary dimension, where Alice and Bob measure generalized versions of the *X* and *Z* bases. This protocol has been implemented, for example, in ref. 18 using orbital angular momentum. Taking *Z* as the standard basis {|*j*〉}, Alice's *X* basis can be taken as the Fourier transform {*F*|*j*〉}, where


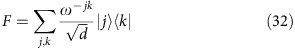


is the Fourier matrix, with *ω*=*e*^2*πi*/*d*^, and for simplicity we choose Alice's and Bob's dimension to be equal: *d*_*A*_=*d*_*B*_=*d*. Bob's *X* basis is set to {F*|*j*〉}, where F* denotes the conjugate of *F* in the standard basis.

Suppose that Alice and Bob observe that their error rates in *Z* and *X* are identical. The theoretical key rates^8^,^17^ for the cases *d*=6, 8, 10 are shown as dashed curves in Fig. 3, while our numerics are shown as circular dots. Clearly there is perfect agreement with the theory.

For our numerics we employ the same constraints as used for BB84 in equation (21)–(24), but generalized to higher *d*. We again emphasize that the calculation of Θ here is very efficient and can easily handle higher dimension. This is because the number of parameters one is optimizing over is independent of dimension—equal to the number of constraints, which in this case is 3. This is in sharp contrast to the primal problem in equation (7), where the number of parameters is *d*^4^, which would be 10,000 for *d*=10.

### *n* MUBs example

**Figure 4:**
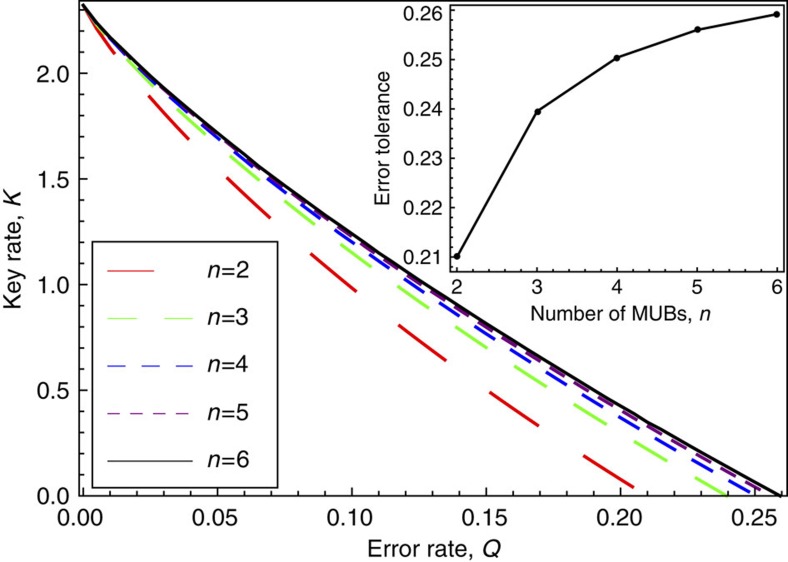
Protocol where Alice and Bob each use *n* MUBs. The key rate is plotted for various *n*∈{2, 3, 4, 5, 6} and for *d*_*A*_=*d*_*B*_=5. This is an unstructured protocol, since for intermediate values of *n* the symmetry group and hence the key rate is unknown. However, our numerics provides the dependence of key rate on error rate for any *n*, as shown. The inset shows the error tolerance—the smallest error rate that makes the key rate vanish—as a function of *n*. Note that the largest jump in the error tolerance occurs from *n*=2 to *n*=3.

A simple generalization of the above protocols is to consider a set of *n* MUBs in dimension *d*. For example, in prime power dimensions there exist explicit constructions for sets of *n* MUBs with 2<*n*<*d*+1 (ref. 35). Consisder a protocol where we fix the set of *n* MUBs, and in each round, Alice and Bob each measure their *d* dimensional system in one basis chosen from this set. For general *n* the symmetry group is not known for this protocol^8^, so one can consider it an unstructured protocol. Indeed, only for the special cases *n*=2 and *n*=*d*+1 do we have analytical formulas for the key rate^8^. Nevertheless it is straightforward to apply our numerics to this protocol for any *n*. Our results are shown in Fig. 4 for *d*=5. To obtain these curves we only need three constraints, which are analogous to equation (29)–(31), but generalized such that 〈*E*_*XY*_〉 is replaced by the average error rate in all *n*−1 bases, excluding the basis used for key generation (the *Z* basis).

Interestingly, Fig. 4 shows that just adding one basis, going from *n*=2 to *n*=3, gives a large jump in the key rate, whereas there are diminishing returns as one adds more bases. This can be seen in the inset of Fig. 4, which plots the error tolerance (that is, the value of *Q* for which the key rate goes to zero) as a function of *n*. We have seen similar behaviour for other *d* besides *d*=5. After completion of this work, an analytical formula for *n*=3 was discovered^36^, and we have verified that it agrees perfectly with our numerics.

In Supplementary Note 3, we analytically prove the following.

*Proposition 2:* Our numerical results are perfectly tight for the protocols discussed in Figs 1, 3 and 4. That is, for these protocols, our optimization exactly reproduces the primal optimization (equation (7)).

Note that this observation implies that key rate for protocols involving *n* MUBs (as in Fig. 4) is now known; namely it is given by our numerical optimization.

### Arbitrary angle between bases example

**Figure 5:**
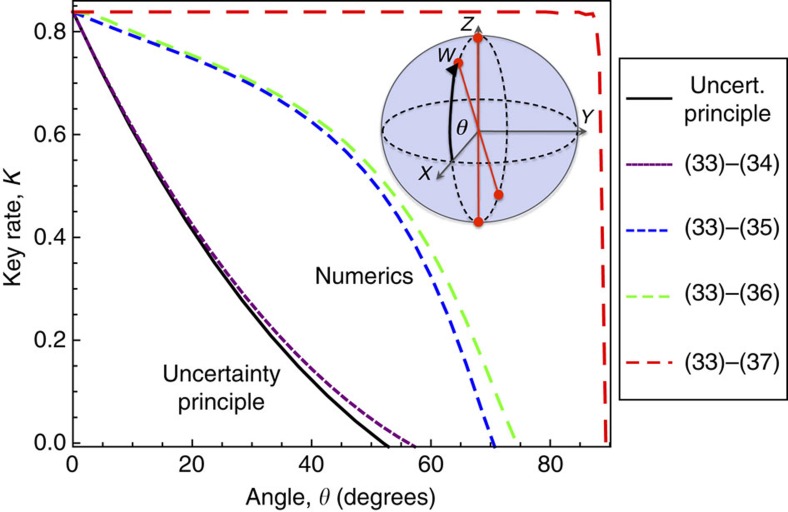
Protocol where Alice and Bob each measure *Z* or *W*. Here *Z* is the standard basis and the *W* basis is rotated by an angle *θ* away from the *X* basis. The key rate versus *θ* is shown with the error rate set to *Q*=0.01. Our numerics give a hierarchy of four lower bounds on the key rate, corresponding to adding in additional constraints from (33)–(37). All of our bounds are tighter than the bound obtained from the entropic uncertainty principle. The plot indicates that the uncertainty principle gives a dramatically pessimistic key rate, much lower than the true key rate of the protocol.

While MUBs are a special case, our approach can handle arbitrary angles between the different measurements or signal states. For example, we consider a simple qubit protocol^37^ where Alice and Bob each measure either the *Z* or *W* basis, where *W* is rotated by an angle *θ* away from the ideal *X* basis. This protocol provides the opportunity to compare our numerical approach to an analytical approach based on the entropic uncertainty principle, introduced in refs 22, 23. This is the state-of-the-art method for lower bounding the key rate. So for comparison, Fig. 5 plots the bound obtained from the entropic uncertainty principle for bases *Z* and *W*.

We apply our numerical approach with the constraints:


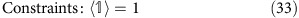



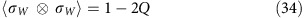



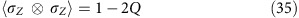



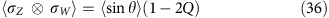



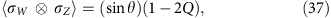


where *σ*_*Z*_ and *σ*_*W*_ are the Pauli operators associated with the *Z* and *W* bases. Figure 5 plots a hierarchy of lower bounds obtained from gradually adding in more of the constraints in equation (33)–(37). As the plot shows, we already beat the entropic uncertainty principle with only the first two constraints. Furthermore, adding in all these constraints gives a markedly higher bound, showing the uncertainty principle gives highly pessimistic key rates for this protocol. From an experimental perspective, Fig. 5 is reassuring, in that small variations in *θ* away from the ideal BB84 protocol (*θ*=0) have essentially no effect on the key rate. Figure 5 also highlights the fact that our approach allows us to systematically study the effect on the key rate of Alice and Bob using more or less of their data. In this example, we see that it is useful to keep data that one will usually discard in the sifting step of the protocol.

### B92 example

Next we consider the B92 protocol^19^, which is a simple, practical and unstructured protocol. It nicely illustrates our framework because it is inherently a prepare-and-measure protocol and it involves post-selection. In the protocol, Alice sends one of two non-orthogonal states {|*φ*_0_〉, |*φ*_1_〉} to Bob. Since the Bloch-sphere angle *θ* between the two states is arbitrary, with 〈*φ*_0_|*φ*_1_〉=cos(*θ*/2), the protocol is unstructured. Bob randomly (with equal probability) measures either in basis 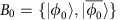 or basis 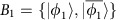, where 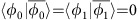. If Bob gets outcome 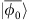 or 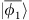, then he publicly announces ‘pass', and he assigns a bit value of 1 or 0, respectively, to his key. Otherwise, Bob announces ‘fail' and they discard the round.

**Figure 6:**
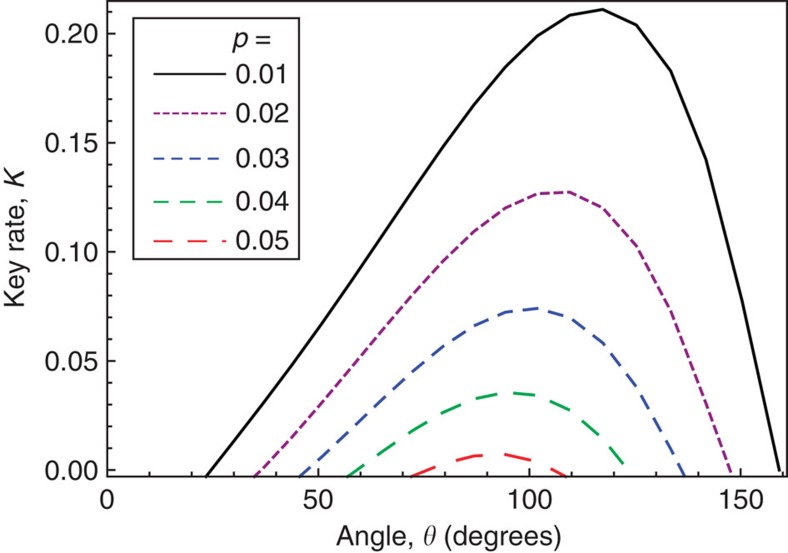
The B92 protocol. The key rate (in bits per photon sent by Ailce) is plotted versus the Bloch-sphere angle between the two signal states. Curves are shown for various values of the depolarizing probability *p*.

A detailed description of the constraints we employed for B92 can be found in Supplementary Note 4. Our numerical results are shown in Fig. 6. Figure 6 shows that the optimal angle for maximizing key rate depends on the depolarizing noise *p*, although small deviations ±5° from the optimal angle do not affect the key rate much.

Our results give higher key rates for B92 than refs 20 and 21, which respectively predicted positive key rates for *p*<0.034 and *p*<0.048, while we predict it for *p*<0.053. On the other hand, ref. 38 directly solved the primal problem equation (7) for B92 by brute-force numerics, and achieves positive key rate for *p*<0.065. We have verified that the gap between our results and those of ref. 38 is due to the looseness of our usage of the Golden-Thompson inequality (equation (60) in the Methods section). However, ref. 38 only showed a plot for *p*⩾0.046, noting that the numerical optimization for the primal problem did not converge for smaller *p* values. This highlights a benefit of going to the dual problem, in that we have no trouble with obtaining the full dependence on *p*.

## Discussion

In conclusion, we address one of the main outstanding problems in QKD theory: how to calculate key rates for arbitrary protocols. Our main result is a numerical method for lower-bounding key rates that is both efficient and reliable. It is reliable in the sense that, by reformulating the problem as a maximization, every solution that one's computer outputs is an achievable key rate. It is efficient in the sense that we have reduced the number of parameters in the optimization problem from 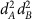 down to the number of experimental constraints, which in some cases is independent of dimension.

The motivation for our work is twofold. First, experimental imperfections tend to break symmetries, so theoretical techniques that exploit symmetries do not apply. Hence, there is no general method currently available for calculating the effect of imperfections on the key rate. Second, it is interesting to ask whether protocols that are intentionally designed to lack symmetry might outperform the well-known symmetric protocols. Such a question cannot be posed without a method for calculating key rates for unstructured protocols. Just to give an example where the key rate is currently unknown, we plan to apply our approach to protocols where a small, discrete set of coherent states are the signal states and information is encoded in the phase^39^.

We envision that our method could be a standard tool for QKD researchers. In future work we hope to extend our approach to the finite-key scenario. Indeed, the optimization problem we solve is closely related to one appearing in finite-key analysis^24^.

## Methods

### Outline

Here we prove our main result, Theorem 1. Our proof relies on several technical tools. First is the notion of the duality of optimization, that is, transforming the primal problem to its dual problem. Second, we employ several entropic identities to simplify the dual problem. Third, we use a recent, important result from ref. 40 that solves a relative entropy optimization problem.

For readability, we prove Theorem 1 here for the special case where the key map POVM *Z*_*A*_=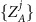 is a projective measurement, that is, where the 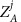 are projectors (of arbitrary rank). We postpone the proof for arbitrary POVMs to Supplementary Note 5.

### The primal problem

First we rewrite equation (7) as:


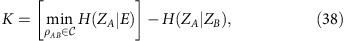


noting that the second term in equation (38), *H*(*Z*_*A*_|*Z*_*B*_), will be determined experimentally and hence can be pulled out of the optimization. We remark that, simply for illustration purposes we used Fano's inequality to upper-bound *H*(*Z*_*A*_|*Z*_*B*_) in our figures; however, in practice *H*(*Z*_*A*_|*Z*_*B*_) would be directly calculated from the data.

Since we only need to optimize the first term, we redefine the primal problem as


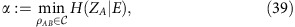


and note that we can take *E* to be a purifying system of *ρ*_*AB*_, since that gives Eve the most information. Next we use a result for tripartite pure states 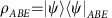 from refs 41, 42 that relates the conditional entropy to the relative entropy:


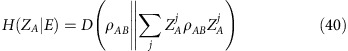


where the relative entropy is defined by


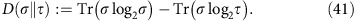


We remark that the joint convexity of the relative entropy implies that the right-hand side of equation (40) is a convex function of *ρ*_*AB*_. (See ref. 43 for an alternative proof of convexity.) Because of this, and the fact that the constraints in equation (5) are linear functions of *ρ*_*AB*_, equation (39) is a convex optimization problem^16^.

It is interesting to point out the connection to coherence^44^. For some set of orthogonal projectors 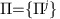 that decompose the identity, 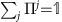, the coherence (sometimes called relative entropy of coherence) of state *ρ* is defined as^44^:


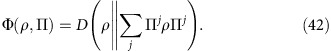


Rewriting the primal problem in terms of coherence gives


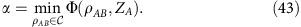


Hence, we make the connection that calculating the secret key rate is related to optimizing the coherence.

This observation is important since the coherence is a continuous function of *ρ* (Supplementary Note 6). This allows us to argue in Supplementary Note 6 that our optimization problem satisfies the strong duality criterion^16^, which means that the solution of the dual problem is precisely equal to that of primal problem.

### The dual problem

Now we transform to the dual problem. Due to a pesky factor of ln(2), it is useful to rescale the primal problem as follows:


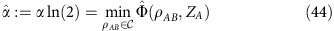


where, henceforth, we generally use the notation 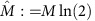, for any quantity *M*. The dual problem^16^ of equation (44) is given by the following unconstrained optimization:


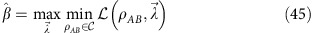


where 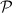 is the set of positive semidefinite operators:


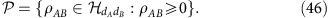


Here the Lagrangian is given by


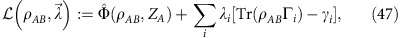


where the 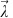
={*λ*_*i*_} are Lagrange multipliers. Strong duality implies that


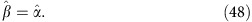


In what follows, we go through several steps to simplify the dual problem. It helps to first state the following lemma from refs 42, 45.

*Lemma 3:* For any ρ and 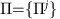, the coherence can be rewritten as


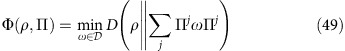


where 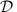 is the set of density operators.

Hence, we have


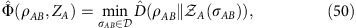


where we define the quantum channel 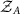 whose action on an operator *O* is given by


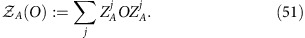


Next, we interchange the two minimizations in (45)


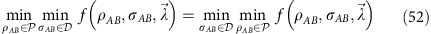


where


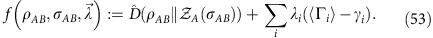


40 solved a relative entropy optimization problem, a special case of which is our problem:


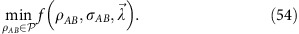


From ref. 40, the unique solution of equation (54) is


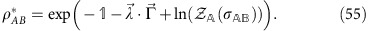


Inserting equation (55) into equation (53) gives the optimal value:


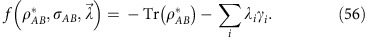


In summary, the dual problem becomes


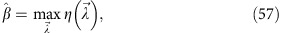


with


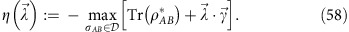


### A lower bound

We can obtain a simple lower bound on *η*(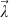) as follows. The Golden–Thompson inequality states that


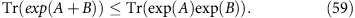


Applying this inequality gives:


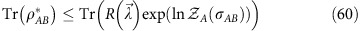



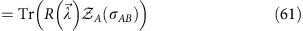



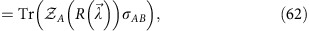


where 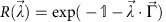 was defined in equation (10). Next, note that


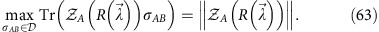


Hence, we arrive at our final result


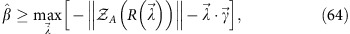


where the right-hand side is denoted as Θ in Theorem 1.

### Data availability

The authors declare that the data supporting the findings in this study are available within the article.

## Additional information

**How to cite this article:** Coles, P. J. *et al*. Numerical approach for unstructured quantum key distribution. *Nat. Commun.* 7:11712 doi: 10.1038/ncomms11712 (2016).

## Supplementary Material

Supplementary Information Supplementary Notes 1-6 and Supplementary References.

## References

[b1] Campagna M. . Quantum Safe Cryptography and Security European Telecommunications Standards Institute (2015).

[b2] Wiesner S. Conjugate coding. ACM SIGACT News 15, 78–88 (1983).

[b3] Bennett C. H. & Brassard G. in *International Conference on Computers, Systems & Signal Processing* 175–179 (Bangalore, India, 1984).

[b4] Scarani V. . The security of practical quantum key distribution. Rev. Mod. Phys. 81, 1301–1350 (2009).

[b5] Lo H.-K., Curty M. & Tamaki K. Secure quantum key distribution. Nat. Photon. 8, 595–604 (2014).

[b6] Sasaki M. . Field test of quantum key distribution in the Tokyo QKD Network. Opt. Express 19, 10387–10409 (2011). 21643295 10.1364/OE.19.010387

[b7] Wang S. . Field and long-term demonstration of a wide area quantum key distribution network. Opt. Express 22, 329–342 (2014). 10.1364/OE.22.021739 25321550

[b8] Ferenczi A. & Lütkenhaus N. Symmetries in quantum key distribution and the connection between optimal attacks and optimal cloning. Phys. Rev. A 85, 052310 (2012).

[b9] Bruss D. Optimal eavesdropping in quantum cryptography with six states. Phys. Rev. Lett. 81, 3018–3021 (1998). 10.1103/PhysRevLett.88.127901 11909501

[b10] Gottesman D., Lo H.-K., Lütkenhaus N. & Preskill J. Security of quantum key distribution with imperfect devices. Quant. Inf. Comput. 4, 325–360 (2004).

[b11] Fung C.-H. F. & Lo H.-K. Security proof of a three-state quantum-key-distribution protocol without rotational symmetry. Phys. Rev. A 74, 042342 (2006).

[b12] Maroy O., Lydersen L. & Skaar J. Security of quantum key distribution with arbitrary individual imperfections. Phys. Rev. A 82, 032337 (2010).

[b13] Woodhead E. Quantum cloning bound and application to quantum key distribution. Phys. Rev. A 88, 012331 (2013).

[b14] Tamaki K., Curty M., Kato G., Lo H.-K. & Azuma K. Loss-tolerant quantum cryptography with imperfect sources. Phys. Rev. A 90, 052314 (2014).

[b15] Devetak I. & Winter A. Distillation of secret key and entanglement from quantum states. Proc. R. Soc. A 461, 207–235 (2005).

[b16] Boyd S. & Vandenberghe L. Convex Optimization Cambridge University Press (2004).

[b17] Sheridan L. & Scarani V. Security proof for quantum key distribution using qudit systems. Phys. Rev. A 82, 030301 (2010).

[b18] Mafu M. . Higher-dimensional orbital-angular-momentum-based quantum key distribution with mutually unbiased bases. Phys. Rev. A 88, 032305 (2013).

[b19] Bennett C. H. Quantum cryptography using any two nonorthogonal states. Phys. Rev. Lett. 68, 3121–3124 (1992). 10045619 10.1103/PhysRevLett.68.3121

[b20] Tamaki K. & Lütkenhaus N. Unconditional security of the Bennett 1992 quantum key-distribution protocol over a lossy and noisy channel. Phys. Rev. A 69, 032316 (2004).

[b21] Renner R., Gisin N. & Kraus B. Information-theoretic security proof for quantum-key-distribution protocols. Phys. Rev. A 72, 012332 (2005). 10.1103/PhysRevLett.95.080501 16196841

[b22] Berta M., Christandl M., Colbeck R., Renes J. M. & Renner R. The uncertainty principle in the presence of quantum memory. Nat. Phys. 6, 659–662 (2010).

[b23] Tomamichel M., Ci C., Lim W., Gisin N. & Renner R. Tight finite-key analysis for quantum cryptography. Nat. Commun. 3, 634 (2012). 22252558 10.1038/ncomms1631 PMC3274703

[b24] Scarani V. & Renner R. Quantum cryptography with finite resources: Unconditional security bound for discrete-variable protocols with one-way postprocessing. Phys. Rev. Lett. 100, 200501 (2008). 18518517 10.1103/PhysRevLett.100.200501

[b25] Sano Y., Matsumoto R. & Uyematsu T. Secure key rate of the BB84 protocol using finite sample bits. J. Phys. A 43, 2677–2681 (2010).

[b26] Renner R. *Security of Quantum Key Distribution* (PhD Thesis, ETH Zurich (2005).

[b27] Renner R. Symmetry of large physical systems implies independence of subsystems. Nat. Phys. 3, 645–649 (2007).

[b28] Christandl M., König R. & Renner R. Postselection technique for quantum channels with applications to quantum cryptography. Phys. Rev. Lett. 102, 020504 (2009). 19257257 10.1103/PhysRevLett.102.020504

[b29] Bennett C., Brassard G. & Mermin N. Quantum cryptography without Bell's theorem. Phys. Rev. Lett. 68, 557–559 (1992). 10045931 10.1103/PhysRevLett.68.557

[b30] Lo H.-K., Ma X. & Chen K. Decoy state quantum key distribution. Phys. Rev. Lett. 94, 230504 (2005). 16090452 10.1103/PhysRevLett.94.230504

[b31] Cao Z., Zhang Z., Lo H.-K. & Ma X. Discrete-phase-randomized coherent state source and its application in quantum key distribution. New J. Phys. 17, 053014 (2015).

[b32] Lo H.-K., Curty M. & Qi B. Measurement-device-independent quantum key distribution. Phys. Rev. Lett. 108, 130503 (2012). 22540686 10.1103/PhysRevLett.108.130503

[b33] Gottesman D. & Lo H. K. Proof of security of quantum key distribution with two-way classical communications. IEEE Trans. Inf. Theory 49, 457–475 (2003).

[b34] Lo H.-K., Chau H. F. & Ardehali M. Efficient quantum key distribution scheme and a proof of its unconditional security. J. Cryptol. 18, 133–165 (2004).

[b35] Bandyopadhyay S., Boykin P. O., Roychowdhury V. & Vatan F. A new proof for the existence of mutually unbiased bases. Algorithmica 34, 512–528 (2001).

[b36] Bradler K., Mirhosseini M., Fickler R., Broadbent A. & Boyd R. Finite-key security analysis for multilevel quantum key distribution. Preprint at http://arxiv.org/abs/1512.05447 (2015).

[b37] Matsumoto R. & Watanabe S. Narrow basis angle doubles secret key in the BB84 protocol. J. Phys. A 43, 145302 (2010).

[b38] Matsumoto R. in *Proceedings of IEEE International Symposium on Information Theory*, 351-353 (2013).

[b39] Lo H. & Preskill J. Security of quantum key distribution using weak coherent states with nonrandom phases. Quant. Inf. Comput. 7, 431–458 (2007).

[b40] Zorzi M., Ticozzi F. & Ferrante A. Minimum relative entropy for quantum estimation: Feasibility and general solution. IEEE Trans. Inf. Theory 60, 357–367 (2014).

[b41] Coles P. J., Yu L., Gheorghiu V. & Griffiths R. B. Information-theoretic treatment of tripartite systems and quantum channels. Phys. Rev. A 83, 062338 (2011).

[b42] Coles P. J. Unification of different views of decoherence and discord. Phys. Rev. A 85, 042103 (2012).

[b43] Watanabe S., Matsumoto R. & Uyematsu T. Tomography increases key rates of quantum-key-distribution protocols. Phys. Rev. A 78, 042316 (2008).

[b44] Baumgratz T., Cramer M. & Plenio M. B. Quantifying coherence. Phys. Rev. Lett. 113, 140401 (2014). 25325620 10.1103/PhysRevLett.113.140401

[b45] Modi K., Paterek T., Son W., Vedral V. & Williamson M. Unified view of quantum and classical correlations. Phys. Rev. Lett. 104, 080501 (2010). 20366919 10.1103/PhysRevLett.104.080501

