# Supplementary material for: Numerical approach for unstructured quantum key distribution
Source: Nat Commun. 2016 May 20;7:11712. doi: 10.1038/ncomms11712 (PMC4876458; doi:10.1038/ncomms11712)
Supplement: Supplementary Information — Supplementary Notes 1-6 and Supplementary References. [file ncomms11712-s1.pdf]

SUPPLEMENTARY NOTE 1: MDI QKD

In the Results section, we outlined our framework for handling MDI QKD protocols. Here we elaborate on this framework, and we also give more details on the example calculation shown in Fig. 2.

Framework for MDI QKD (continued). Our framework considers the tripartite state ρ_{ABM}, where A and B are respectively the systems held by Alice and Bob in the source-replacement scheme, and M is the classical register that stores the outcome of the measurement performed by the untrusted node. Let us elaborate on the origin of ρ_{ABM}. Recall that, in the source-replacement scheme, Alice prepares a bipartite entangled state of the form

$$|\psi_{AA'}\rangle = \sum_j \sqrt{p_j}|j\rangle|\phi_j\rangle, \tag{1}$$

and in the MDI scenario, Bob prepares a similar state

$$|\psi_{BB'}\rangle = \sum_j \sqrt{p_j}|j\rangle|\phi_j\rangle\,. \tag{2}$$

Hence, the initial state (prior to the action of Eve) is

$$\rho^{(0)}_{AA'BB'} := |\psi_{AA'}\rangle\langle\psi_{AA'}| \otimes |\psi_{BB'}\rangle\langle\psi_{BB'}|\,. \tag{3}$$

For notational convenience, it is helpful to permute the order of the subsystems, as follows

$$\tilde{\rho}^{(0)}_{ABA'B'} := \mathcal{F}\Big(\rho^{(0)}_{AA'BB'}\Big)\,, \tag{4}$$

where $\mathcal{F}$ is the quantum channel that switches the ordering of subsystems A' and B.

Now note that Eve only has access to $A'B'$ and not AB. Likewise the untrusted node performs a measurement only on $A'B'$, while A and B remain respectively in Alice's and Bob's laboratories. We combine the action of Eve together with the action of the untrusted measurement, and model it as a single quantum channel $\mathcal{E}$ that maps $A'B' \to M$, where M is a classical register. That is, we obtain the state

$$\rho_{ABM} = \Big(\mathcal{I}_{AB} \otimes \mathcal{E}\Big)\Big(\tilde{\rho}^{(0)}_{ABA'B'}\Big)\,, \tag{5}$$

where $\mathcal{I}_{AB}$ is the identity channel on AB. We apply our numerical approach to the state ρ_{ABM} in Supplementary Eq. (5). The beauty of the MDI protocol is that we do not need to consider the process of how we arrived at the state ρ_{ABM}, i.e., we do not need to discuss the details of the channel $\mathcal{E}$. We only need to specify the experimental constraints on ρ_{ABM}, which we stated in the Results section (although we repeat them here for convenience),

$$\mathrm{Tr}\left[\rho_{ABM}(|j\rangle\langle j| \otimes |k\rangle\langle k| \otimes |m\rangle\langle m|)\right] = \gamma_{jkm}\,. \tag{6}$$

In addition, we also enforce constraints that fix the form of the marginal ρ_{AB}, which has the form

$$\rho_{AB} = \rho_A \otimes \rho_B = \left(\sum_{j,k} \sqrt{p_j p_k}\langle\phi_k|\phi_j\rangle|j\rangle\langle k|\right) \otimes \left(\sum_{j,k} \sqrt{p_j p_k}\langle\phi_k|\phi_j\rangle|j\rangle\langle k|\right)\,. \tag{7}$$

One could also add constraints that enforce that M is a classical system. However, we choose not to do this for the following reason. The worst-case scenario, i.e., the scenario that gives Eve the most information, corresponds to M being classical, and hence the key rate is not improved by enforcing the classicality of M. We state this in the following lemma.

Supplementary Lemma 1: Let $\{|m\rangle\}$ be the standard basis for system M, and let $\mathcal{M}$ be the quantum channel that diagonalizes (i.e., decoheres) system M in this basis. That is, $\mathcal{M}(O) = \sum_m |m\rangle\langle m|O|m\rangle\langle m|$ for any operator O. Consider a set of constraints C on ρ_{ABM} and let $\mathcal{C}$ denote the set of density operators ρ_{ABM} that satisfy C. Suppose that the constraints C do not preclude M from being decohered in the standard basis, i.e., if $\rho_{ABM} \in \mathcal{C}$, then $(\mathcal{I}_{AB} \otimes \mathcal{M})(\rho_{ABM}) \in \mathcal{C}$. Define the set

$$\mathcal{C}_{\mathcal{M}} := \{\rho_{ABM} \in \mathcal{C} : (\mathcal{I}_{AB} \otimes \mathcal{M})(\rho_{ABM}) = \rho_{ABM}\}\,. \tag{8}$$

In other words, $\mathcal{C}_{\mathcal{M}} \subseteq \mathcal{C}$ is the set of states in $\mathcal{C}$ that are diagonal in the standard basis on M. Then, Eve's ignorance about Alice's key is the same regardless of whether we impose that M is decohered in the standard basis, i.e.,

$$\min_{\rho_{ABM}\in\mathcal{C}} H(Z_A|E) = \min_{\rho_{ABM}\in\mathcal{C}_{\mathcal{M}}} H(Z_A|E)\,. \tag{9}$$

Proof. For notational simplicity we drop the subscript A from Z_A in what follows. Since $\mathcal{C}_{\mathcal{M}} \subseteq \mathcal{C}$, then we obviously have

$$\min_{\rho_{ABM}\in\mathcal{C}} H(Z|E) \leqslant \min_{\rho_{ABM}\in\mathcal{C}_{\mathcal{M}}} H(Z|E)\,, \tag{10}$$

so we just need to show the inequality in the opposite direction. In particular we will show that for each state in $\mathcal{C}$ there is a corresponding state in $\mathcal{C}_{\mathcal{M}}$ where Eve's ignorance is lower. Let $\rho_{ABM} \in \mathcal{C}$, then

$$\tilde{\rho}_{ABM} := (\mathcal{I}_{AB} \otimes \mathcal{M})(\rho_{ABM}) \in \mathcal{C}_{\mathcal{M}}\,. \tag{11}$$

Let E and $\tilde{E}$ be purifying systems for ρ_{ABM} and $\tilde{\rho}_{ABM}$, respectively. Then the states

$$\sigma_{ZZ'ABME} := (V \otimes \mathbb{1}_{BME})\rho_{ABME}(V^\dagger \otimes \mathbb{1}_{BME})\,, \quad \text{and} \tag{12}$$

$$\tilde{\sigma}_{ZZ'ABM\tilde{E}} := (V \otimes \mathbb{1}_{BM\tilde{E}})\tilde{\rho}_{ABM\tilde{E}}(V^\dagger \otimes \mathbb{1}_{BM\tilde{E}}) \tag{13}$$

are pure states. Here, V is an isometry that maps $A \to ZZ'A$, defined by

$$V := \sum_j |j\rangle_Z \otimes |j\rangle_{Z'} \otimes \sqrt{Z_A^j}\,, \tag{14}$$

where the set $\{Z_A^j\}$ forms a POVM (Alice's key-map POVM).

Let us take a moment to clarify the meaning of the conditional entropy $H(Z|E)$. Note that, by convention, when we casually refer to $H(Z|E)$ for the state ρ_{ABME}, we precisely mean the conditional von Neumann entropy of the state $\sigma_{ZZ'ABME}$, which we denote $H(Z|E)_\sigma$. Typically one refers to $\sigma_{ZZ'ABME}$ as the post-measurement state associated with a given (pre-measurement) state ρ_{ABME}. Likewise $H(Z|\tilde{E})$ for the state $\tilde{\rho}_{ABM\tilde{E}}$ actually refers to the conditional von Neumann entropy of $\tilde{\sigma}_{ZZ'ABM\tilde{E}}$ denoted by $H(Z|\tilde{E})_{\tilde{\sigma}}$.

The duality [1] of the von Neumann entropy says that $H(A|B)_\tau = -H(A|C)_\tau$ for any tripartite pure state τ_{ABC}. Applying this duality relation to the pure state $\sigma_{ZZ'ABME}$ gives

$$\begin{aligned} H(Z|E)_\sigma &= -H(Z|Z'ABM)_\sigma & (15)\\ &\geqslant -H(Z|Z'ABM)_{\tilde{\sigma}} & (16)\\ &= H(Z|\tilde{E})_{\tilde{\sigma}}\,, & (17) \end{aligned}$$

where the inequality is due to the data-processing inequality, i.e., acting with channel $\mathcal{M}$ on M can never reduce the entropy. Hence we have shown that Eve's ignorance for the state $\tilde{\rho}_{ABM\tilde{E}}$ is not larger than her ignorance for the state ρ_{ABME}, which is the desired result. □

Example: MDI QKD with BB84 states. Here we elaborate on how we obtain the data in Fig. 2. To obtain this data, we consider the most common MDI protocol, where Alice and Bob each prepare and send the BB84 signal states $\{|0\rangle, |1\rangle, |+\rangle, |-\rangle\}$ with probabilities $p_z/2$ and $(1-p_z)/2$ respectively for the Z- and X-basis states. For simplicity we consider a protocol that does not do sifting and distills key out of both the Z- and X-bases. This corresponds to choosing the key map as

$$\text{Key-map POVM: } Z_A = \{|0\rangle\langle 0| + |2\rangle\langle 2|, |1\rangle\langle 1| + |3\rangle\langle 3|\}\,, \tag{18}$$

where Alice's source-replacement state from Eq. (12) is

$$|\psi_{AA'}\rangle = \sqrt{p_z/2}\,(|0\rangle|0\rangle + |1\rangle|1\rangle) + \sqrt{(1-p_z)/2}\,(|2\rangle|+\rangle + |3\rangle|-\rangle)\,. \tag{19}$$

To obtain large key rates we employ biased basis choices [2], i.e., $p_z = 1-\epsilon$ with $0 < \epsilon \ll 1$. As noted above, we impose the correlation constraints in Supplementary Eq. (6) as well as constraints that fix the form of the marginals ρ_A and ρ_B, Supplementary Eq. (7). It is encouraging that our numerics reproduce the known theoretical curve [3], as shown in Fig. 2.

SUPPLEMENTARY NOTE 2: ARBITRARY POST-SELECTION

In the Results section we discussed a method for transforming the constraints on ρ_{AB} to constraints on the post-selected state $\mathcal{G}(\rho_{AB})$ for the special case where $\mathcal{G}$ has a CP inverse. We now generalize the method to transform the constraints for any CP map $\mathcal{G}$.

The idea is to view the space of Hermitian operators as a vector space, and to partition the space into basis vectors whose coefficients are fixed by the constraints and those whose coefficients are free. Namely, we apply this view to the image space under post-selection, as follows.

By applying the Gram-Schmidt process to the measurement operators $\{\Gamma_i\}$, we can write the constraints on ρ_{AB} equivalently as $\mathrm{Tr}(\rho_{AB}\Delta_i) = \delta_i$ where the $\{\Delta_i\}$ are orthonormal under the Frobenius inner product $\langle A, B\rangle = \mathrm{Tr}(A^\dagger B)$. We extend this to an orthonormal basis $\{\Delta_i\} \cup \{\Xi_j\}$ of the Hermitian operator space. Note that here, and in what follows, we take the basis elements to be Hermitian.

For clarity, in what follows we refer to the observation-based constraints on ρ_{AB}, which are generally of the form $\mathrm{Tr}(\rho_{AB}\Gamma_i) = \gamma_i$, as trace constraints. This is to distinguish them from the constraints on ρ_{AB} due to its positivity. Now define $(\vec{\delta})_i = \delta_i$ and $(\vec{\Delta})_i = \Delta_i$, and similarly for $\vec{\xi}$ and $\vec{\Xi}$. Then any ρ_{AB} satisfying the trace constraints is of the form:

$$\rho_{AB} = \vec{\delta} \cdot \vec{\Delta} + \vec{\xi} \cdot \vec{\Xi} = \rho_0 + \vec{\xi} \cdot \vec{\Xi}, \tag{20}$$

for any $\vec{\xi}$. Note that the requirement that $\rho_{AB} \geqslant 0$ will constrain the possible values of $\vec{\xi}$, but for now we only consider the trace constraints. For later convenience, we have defined the (not necessarily positive semidefinite) operator $\rho_0 := \vec{\delta} \cdot \vec{\Delta}$.

Acting linearly with $\mathcal{G}$, we find that the image of the set of ρ_{AB} satisfying the trace constraints is

$$\mathcal{G}(\rho_{AB}) = \mathcal{G}(\rho_0) + \vec{\xi} \cdot \mathcal{G}(\vec{\Xi}). \tag{21}$$

Now, let $\{\Upsilon_m\}$ be an orthonormal basis for the space spanned by the operators $\{\mathcal{G}(\Xi_j)\}$, which can again be found by the Gram-Schmidt process. We can extend this to an orthonormal basis $\{\Upsilon_m\} \cup \{\Omega_n\}$ of the image of the original Hilbert space under $\mathcal{G}$, i.e., $\mathcal{G}(\mathcal{H}_{AB})$. In practice, this can be done by performing Gram-Schmidt again on $\{\Upsilon_m\} \cup \{\mathcal{G}(\Delta_i)\}$, which will leave the Υ_m unchanged. The $\{\Omega_n\}$ are the operators of interest. We can calculate the coefficients ω_n:

$$\mathrm{Tr}(\mathcal{G}(\rho_0)\Omega_n) = \omega_n. \tag{22}$$

With the basis decomposition $\mathcal{G}(\rho_{AB}) = \vec{\omega} \cdot \vec{\Omega} + \vec{v} \cdot \vec{\Upsilon}$ for some $\vec{v}$, it can be shown that the trace constraints on ρ_{AB} do not constrain $\vec{v}$ whatsoever. On the other hand, the coefficients in $\vec{\omega}$ are exactly determined in Supplementary Eq. (22). Thus, the trace constraints are exactly converted from ρ_{AB} to $\mathcal{G}(\rho_{AB})$ according to:

$$\{\mathrm{Tr}(\rho_{AB}\Gamma_i) = \gamma_i\} \Rightarrow \{\mathrm{Tr}(\mathcal{G}(\rho_{AB})\Omega_n) = \omega_n\}. \tag{23}$$

Let us remark that the Hermitian operators in $\mathcal{G}(\mathcal{H}_{AB})$ will be represented as matrices in a possibly larger space $\tilde{\mathcal{H}}_{AB}$, where $\mathcal{G}(\mathcal{H}_{AB}) \subseteq \tilde{\mathcal{H}}_{AB}$. One has the freedom to choose $\tilde{\mathcal{H}}_{AB}$ for a convenient matrix representation of $\mathcal{G}(\mathcal{H}_{AB})$, and hence $\tilde{\mathcal{H}}_{AB}$ is not unique. But if $\tilde{\mathcal{H}}_{AB}$ is strictly larger, i.e., $\mathcal{G}(\mathcal{H}_{AB}) \subset \tilde{\mathcal{H}}_{AB}$, then we must enforce additional trace constraints, to restrict the optimization to $\mathcal{G}(\mathcal{H}_{AB})$. To obtain these additional constraints, complete the orthonormal basis $\{\Upsilon_m\} \cup \{\Omega_n\}$ of $\mathcal{G}(\mathcal{H}_{AB})$ to a basis of $\tilde{\mathcal{H}}_{AB}$ with the additional orthonormal Hermitian operators $\{\Lambda_\ell\}$. The operators $O \in \tilde{\mathcal{H}}_{AB}$ such that $O \in \mathcal{G}(\mathcal{H}_{AB})$ are exactly those that satisfy $\mathrm{Tr}(O\Lambda_\ell) = 0$ for each ℓ. Hence, one can add the constraints $\{\mathrm{Tr}(\mathcal{G}(\rho_{AB})\Lambda_\ell) = 0\}$ to the set in Supplementary Eq. (23). For future convenience, let us define the set of positive operators in $\tilde{\mathcal{H}}_{AB}$ as $\tilde{\mathcal{P}}_{AB} := \{\tilde{\rho}_{AB} \in \tilde{\mathcal{H}}_{AB} : \tilde{\rho}_{AB} \geqslant 0\}$.

With post-selection, the key rate formula in Eq. (1) is applied to the post-selected state $\mathcal{G}(\rho_{AB})/p_{\mathrm{pass}}$, with the measured quantity $p_{\mathrm{pass}} = \mathrm{Tr}(\mathcal{G}(\rho_{AB}))$. (See the remark in the main text where we note that taking Eve's system to purify the post-selected state does not introduce any looseness into our key rate calculation.) In the usual primal optimization, the optimization is taken over ρ_{AB}. However, we can directly reformulate it as an optimization over $\tilde{\rho}_{AB}$ in $\mathcal{G}(\mathcal{P}_{AB})$, the image of $\mathcal{P}_{AB}$ under the post-selection map. Let $\tilde{\mathcal{B}}$ be the set of $\tilde{\rho}_{AB} \in \mathcal{G}(\mathcal{P}_{AB})$ satisfying the

trace constraints $\mathrm{Tr}(\tilde{\rho}_{AB}\Omega_n) = \omega_n$ for each n. Then the primal problem is:

$$\widehat{\alpha} = \min_{\rho_{AB}\in\mathcal{C}} \widehat{\Phi}\left(\frac{\mathcal{G}(\rho_{AB})}{p_{\text{pass}}}, Z_A\right) \tag{24}$$

$$= \min_{\rho_{AB}\in\mathcal{C}} \min_{\sigma_{AB}\in\mathcal{D}} \widehat{D}\left(\frac{\mathcal{G}(\rho_{AB})}{p_{\text{pass}}}\middle|\middle|\mathcal{Z}_A(\sigma_{AB})\right) \tag{25}$$

$$= \frac{\widetilde{\alpha}}{p_{\text{pass}}} - \ln p_{\text{pass}}\,, \tag{26}$$

where

$$\widetilde{\alpha} := \min_{\tilde{\rho}_{AB}\in\tilde{\mathcal{B}}} \min_{\sigma_{AB}\in\mathcal{D}} \widehat{D}\big(\tilde{\rho}_{AB}||\mathcal{Z}_A(\sigma_{AB})\big)\,. \tag{27}$$

To apply our standard optimization algorithm, we need to optimize over a set of all positive semidefinite operators in a Hilbert space. Since $\mathcal{G}$ is a CP map, $\mathcal{G}(\mathcal{P}_{AB}) \subseteq \mathcal{G}(\mathcal{H}_{AB})_+$, where $\mathcal{G}(\mathcal{H}_{AB})_+$ is the set of positive semidefinite operators in $\mathcal{G}(\mathcal{H}_{AB})$. The inclusion need not be with equality, so we have the inequality:

$$\widetilde{\alpha} \geq \min_{\tilde{\rho}_{AB}\in\tilde{\mathcal{C}}} \min_{\sigma_{AB}\in\mathcal{D}} \widehat{D}\big(\tilde{\rho}_{AB}||\mathcal{Z}_A(\sigma_{AB})\big), \tag{28}$$

where $\tilde{\mathcal{C}}$ is the set of $\tilde{\rho}_{AB} \in \mathcal{G}(\mathcal{H}_{AB})_+$ such that $\mathrm{Tr}(\tilde{\rho}_{AB}\Omega_n) = \omega_n$ for each n, or equivalently, the set of $\tilde{\rho}_{AB} \in \tilde{\mathcal{P}}_{AB}$ such that $\mathrm{Tr}(\tilde{\rho}_{AB}\Omega_n) = \omega_n$ for each n and $\mathrm{Tr}(\tilde{\rho}_{AB}\Lambda_\ell) = 0$ for each ℓ. With the reformulation of the optimization problem in Supplementary Eq. (28), we have (at the expense of introducing an inequality) recast the optimization with post-selection into the usual form treated in the Methods section.

However, note that when $\mathcal{G}$ has an inverse $\mathcal{G}^{-1}$ that is CP, Supplementary Eq. (28) is satisfied with equality. This follows from the fact that $\mathcal{G}(\mathcal{P}_{AB}) = \mathcal{G}(\mathcal{H}_{AB})_+$ in this case. This special case was discussed in the Results section. Furthermore, we note that the B92 protocol (see Supplementary Note 4) involves a post-selection map that has a CP inverse. So for that protocol, the step in Supplementary Eq. (28) does not introduce any looseness.

SUPPLEMENTARY NOTE 3: TIGHTNESS FOR PROTOCOLS WITH MUBS

Here we analytically prove Prop. 2. This states that our numerical approach is perfectly tight for the entanglement-based protocols involving MUBs discussed in the main text.

First we note that the only potential source of looseness in our bound is our usage in Eq. (60) of the Golden-Thompson (GT) inequality Eq. (59). The question, then, is under what conditions is Eq. (60) saturated.

A general lemma

We begin by stating a general lemma, which gives a sufficient set of criteria that guarantee our method is tight. Note that these sufficient criteria might not be necessary for tightness.
Supplementary Lemma 2: The GT inequality invoked in Eq. (60) is saturated, and hence our method tight, for a QKD protocol satisfying the following two conditions:

(a) $[\Gamma_i, \Gamma_{i'}] = 0 \;\; \forall\, i, i'$

(b) $\langle e_\ell|\mathcal{Z}_A(|e_k\rangle\langle e_k|)|e_{\ell'}\rangle = 0$ for $\ell \neq \ell'$ and $\forall k$ in a common eigenbasis $\{|e_k\rangle\}$ of all $\{\Gamma_i\}$.

Proof. In general, the GT inequality Eq. (59) is satisfied with equality if and only if the two operators commute. In our case, the saturation of the GT inequality is equivalent to the vanishing of the following commutator

$$\Big[Q(\vec{\lambda}), \ln \mathcal{Z}_A(\sigma^*_{AB})\Big]\,, \tag{29}$$

where $Q(\vec{\lambda}) := -\mathbb{1} - \vec{\lambda}\cdot\vec{\Gamma}$, and where σ^*_{AB} is a maximal eigenvector of

$$T := \mathcal{Z}_A\big(\exp(Q(\vec{\lambda}))\big) = \mathcal{Z}_A\big(R(\vec{\lambda})\big)\,, \tag{30}$$

i.e., an eigenvector of T whose eigenvalue is the largest. In general σ^*_{AB} is not uniquely defined if the maximal eigenvalue is degenerate. However, this issue does not affect the proof below. This is because, for tightness, we only need the GT inequality to be saturated for one particular σ^*_{AB}, i.e., one particular σ_{AB} that achieves the optimization in Eq. (63).

As $\mathcal{Z}_A(\sigma^*_{AB})$ is positive semidefinite, it can be shown that the vanishing of Supplementary Eq. (29), and thus the saturation of the GT inequality, is equivalent to the vanishing of

$$\left[Q(\vec{\lambda}), \mathcal{Z}_A(\sigma^*_{AB})\right] . \tag{31}$$

This follows from the fact that $\ln \mathcal{Z}_A(\sigma^*_{AB})$ and $\mathcal{Z}_A(\sigma^*_{AB}) = \exp(\ln \mathcal{Z}_A(\sigma^*_{AB}))$ are diagonal in the same basis.

Now suppose that conditions (a) and (b) are satisfied. It follows from (a) that the measurement operators $\{\Gamma_i\}$ can be simultaneously diagonalized in an orthonormal eigenbasis $\{|e_k\rangle\}$. The operators $Q(\vec{\lambda})$ and $R(\vec{\lambda}) = \exp(Q(\vec{\lambda}))$ are also diagonal in such a basis.

From condition (b), we note that $\mathcal{Z}_A$ maps an eigenstate $|e_k\rangle\langle e_k|$ to a linear combination of $|e_\ell\rangle\langle e_\ell|$ terms. Let the coefficients of that combination be $b_{k\ell}$ and let the eigenvalues of $R(\vec{\lambda})$ be a_k. Then T is also diagonalizable in the $\{|e_k\rangle\}$ eigenbasis since

$$T = \sum_k a_k \mathcal{Z}_A(|e_k\rangle\langle e_k|) = \sum_\ell \left(\sum_k b_{k\ell} a_k\right) |e_\ell\rangle\langle e_\ell|. \tag{32}$$

Since σ^*_{AB} is a maximal eigenvector of T, and T is diagonal in the $\{|e_k\rangle\}$ basis, then let us choose $\sigma^*_{AB} = |e_m\rangle\langle e_m|$ to correspond to a state $|e_m\rangle$ from this basis. While T may have more than one eigenbasis, we remark that we have the freedom to choose σ^*_{AB} from the $\{|e_k\rangle\}$ basis, since (as noted above) we only need the GT inequality to be saturated for a particular choice of σ^*_{AB}.

We find that Supplementary Eq. (31) vanishes:

$$\left[Q(\vec{\lambda}), \mathcal{Z}_A(\sigma^*_{AB})\right] = \left[Q(\vec{\lambda}), \sum_\ell b_{m\ell} |e_\ell\rangle\langle e_\ell|\right] = \sum_\ell b_{m\ell} \left[Q(\vec{\lambda}), |e_\ell\rangle\langle e_\ell|\right] = 0 \,, \tag{33}$$

and thus the GT inequality is saturated if conditions (a) and (b) are satisfied. □

Specific protocols

We now show that conditions (a) and (b) in Supplementary Lemma 2 are satisfied for the protocols involving MUBs in the main text.

First we define some notation. The generalized Pauli operators in dimension d are

$$\sigma_Z := \sum_j \omega^j |j\rangle\langle j| \tag{34}$$

$$\sigma_X := \sum_j |j+1\rangle\langle j| = F \sigma_Z F^\dagger \,, \tag{35}$$

with $\omega = e^{2\pi i/d}$. From these operators one can construct the Bell basis states $\{|\phi_{q,r}\rangle\}$, i.e., a set of d^2 orthonormal states of the form

$$|\phi_{q,r}\rangle := \mathbb{1} \otimes \sigma_X^q \sigma_Z^r |\phi_{0,0}\rangle, \quad \text{with } q, r \in \{0, ..., d-1\} \,, \tag{36}$$

where

$$|\phi_{0,0}\rangle := \sum_j \frac{1}{\sqrt{d}} |j\rangle|j\rangle \,. \tag{37}$$

Our proof of tightness will proceed by showing that the Γ_i operators of interest are all diagonal in the Bell basis (Supplementary Eq. (36)), and furthermore that the Bell basis satisfies condition (b) in Supplementary Lemma 2. Let us first show the latter, since it will be used repeatedly below.

Supplementary Lemma 3: The Bell basis $\{|\phi_{q,r}\rangle\}$ satisfies condition (b) in Supplementary Lemma 2. That is,

$$\langle\phi_{\ell,m}|\mathcal{Z}_A(|\phi_{q,r}\rangle\langle\phi_{q,r}|)|\phi_{\ell',m'}\rangle = 0, \quad \forall(\ell,m)\neq(\ell',m') \text{ and } \forall(q,r)\,. \tag{38}$$

Proof. In all the protocols under consideration,

$$Z_A = \{|j\rangle\langle j|\}_{j=0}^{d-1} \tag{39}$$

is taken to be the standard basis on system A. Hence we can rewrite the action of the channel $\mathcal{Z}_A$ on some operator O as

$$\begin{aligned}\mathcal{Z}_A(O) &= \sum_j |j\rangle\langle j|O|j\rangle\langle j| &(40)\\ &= \frac{1}{d}\sum_{j,k,k'}\omega^{j(k-k')}|k\rangle\langle k|O|k'\rangle\langle k'| &(41)\\ &= \frac{1}{d}\sum_j \sigma_Z^j O(\sigma_Z^j)^\dagger\,. &(42)\end{aligned}$$

Next note that $|\phi_{0,0}\rangle$ has the property

$$(O\otimes \mathbb{1})|\phi_{0,0}\rangle = (\mathbb{1}\otimes O^T)|\phi_{0,0}\rangle \tag{43}$$

for some operator O, where ^{T} is the transpose in the standard basis. Hence we have

$$\begin{aligned}\mathcal{Z}_A(|\phi_{0,0}\rangle\langle\phi_{0,0}|) &= \frac{1}{d}\sum_j(\sigma_Z^j\otimes\mathbb{1})|\phi_{0,0}\rangle\langle\phi_{0,0}|((\sigma_Z^j)^\dagger\otimes\mathbb{1}) &(44)\\ &= \frac{1}{d}\sum_j(\mathbb{1}\otimes\sigma_Z^j)|\phi_{0,0}\rangle\langle\phi_{0,0}|(\mathbb{1}\otimes(\sigma_Z^j)^\dagger)\,. &(45)\end{aligned}$$

Finally, using the definition in Supplementary Eq. (36), we have

$$\begin{aligned}\mathcal{Z}_A(|\phi_{q,r}\rangle\langle\phi_{q,r}|) &= (\mathbb{1}\otimes\sigma_X^q\sigma_Z^r)\mathcal{Z}_A(|\phi_{0,0}\rangle\langle\phi_{0,0}|)(\mathbb{1}\otimes\sigma_X^q\sigma_Z^r)^\dagger &(46)\\ &= \frac{1}{d}\sum_j(\mathbb{1}\otimes\sigma_X^q\sigma_Z^{r+j})|\phi_{0,0}\rangle\langle\phi_{0,0}|(\mathbb{1}\otimes\sigma_X^q\sigma_Z^{r+j})^\dagger &(47)\\ &= \frac{1}{d}\sum_j|\phi_{q,r+j}\rangle\langle\phi_{q,r+j}|\,. &(48)\end{aligned}$$

Clearly Supplementary Eq. (48) is diagonal in the Bell basis, proving the desired result. □

Therefore, in the specific protocols considered below, we only need to show that the $\{\Gamma_i\}$ operators are diagonal in the Bell basis, to prove tightness of our method.

Two MUBs

First let us consider the protocol discussed in the main text involving only two MUBs in arbitrary dimension d. Here the $\{\Gamma_i\}$ operators are $\{\mathbb{1}, E_Z, E_X\}$, where we write the error operators as $E_Z = \mathbb{1} - C_Z$ and $E_X = \mathbb{1} - C_X$, with

$$\begin{aligned}C_Z &:= \sum_j |j\rangle\langle j|\otimes|j\rangle\langle j| &(49)\\ C_X &:= \sum_j F|j\rangle\langle j|F^\dagger\otimes F^\dagger|j\rangle\langle j|F. &(50)\end{aligned}$$

It suffices to show that C_Z and C_X are diagonal in the Bell basis. First, note that C_Z is d times the quantity in Supplementary Eq. (45), and hence

$$C_Z = \sum_{r=0}^{d-1} |\phi_{0,r}\rangle\langle\phi_{0,r}|, \tag{51}$$

which is obviously diagonal in the Bell basis. Next we write

$$\begin{aligned} C_X &= (F \otimes F^\dagger) C_Z (F^\dagger \otimes F) & (52)\\ &= \sum_r (F \otimes F^\dagger)|\phi_{0,r}\rangle\langle\phi_{0,r}|(F^\dagger \otimes F) & (53)\\ &= \sum_r (F \otimes F^\dagger \sigma_Z^r)|\phi_{0,0}\rangle\langle\phi_{0,0}|(F^\dagger \otimes (\sigma_Z^r)^\dagger F) & (54)\\ &= \sum_r (\mathbb{1} \otimes F^\dagger \sigma_Z^r F)|\phi_{0,0}\rangle\langle\phi_{0,0}|(\mathbb{1} \otimes F^\dagger (\sigma_Z^r)^\dagger F) & (55)\\ &= \sum_r (\mathbb{1} \otimes \sigma_X^{d-r})|\phi_{0,0}\rangle\langle\phi_{0,0}|(\mathbb{1} \otimes (\sigma_X^{d-r})^\dagger) & (56)\\ &= \sum_r |\phi_{r,0}\rangle\langle\phi_{r,0}|\,, & (57) \end{aligned}$$

where Supplementary Eq. (56) used the relation

$$F^\dagger \sigma_Z^r F = (F \sigma_Z^r F^\dagger)^T = (\sigma_X^r)^T = \sigma_X^{d-r}\,. \tag{58}$$

Clearly the final expression for C_X is diagonal in the Bell basis. This proves that our numerical approach is tight for the protocol discussed in Fig. 3 of the main text.

Six-state protocol

The six-state protocol (see Fig. 1) is a qubit protocol involving the operators $\{\Gamma_i\} = \{\mathbb{1}, E_Z, E_X + E_Y\}$. We already showed that E_Z and E_X are diagonal in the Bell basis, so we just need to do the same for E_Y. Note that we can write $E_Y = (\mathbb{1} - \sigma_Y \otimes \sigma_Y)/2$, where $\sigma_Y := -i|0\rangle\langle 1| + i|1\rangle\langle 0|$. So it suffices to show that $\sigma_Y \otimes \sigma_Y$ is diagonal in the Bell basis. This follows from directly computing the action on the four Bell states:

$$\sigma_Y \otimes \sigma_Y(|00\rangle \pm |11\rangle) = -(|11\rangle \pm |00\rangle) \tag{59}$$

$$\sigma_Y \otimes \sigma_Y(|01\rangle \pm |10\rangle) = (|10\rangle \pm |01\rangle)\,. \tag{60}$$

Hence our method is tight for the six-state protocol.

n MUBs

The protocol considered in Fig. 4 involved n MUBs in $d = 5$. These MUBs were chosen based on a construction in Ref. [4]. Namely, in prime dimension, the eigenvectors of the operators

$$\sigma_Z, \sigma_X, \sigma_X\sigma_Z, ..., \sigma_X\sigma_Z^{d-1} \tag{61}$$

form a set of $d+1$ MUBs. In Fig. 4, we considered a subset of size n of the MUBs in Supplementary Eq. (61).

The measurement operators are:

$$\{\Gamma_i\} = \{\mathbb{1}, E_Z, E_X + E_{XZ} + ... + E_{XZ^{n-2}}\} \tag{62}$$

where E_{XZ^k} denotes the error operator for the basis associated with $\sigma_X\sigma_Z^k$.

We already showed above that E_Z and E_X are diagonal in the Bell basis, so it remains to show this for $E_{XZ}, ..., E_{XZ^{n-2}}$. Again let us use the notation

$$\begin{aligned} C_{XZ^k} &:= \mathbb{1} - E_{XZ^k} & (63)\\ &= (H_k \otimes H_k^*) C_Z (H_k \otimes H_k^*)^\dagger\,, & (64) \end{aligned}$$

where H_k is the Hadamard (unitary) matrix that rotates the standard basis to the eigenbasis of $\sigma_X\sigma_Z^k$, and H_k^* denotes its conjugate in the standard basis.

Consider the case where d is an odd prime. Note that the only even prime is $d = 2$ which we already covered above. We restrict to odd primes here, since the following construction applies to them

$$H_k = \sum_{j,j'} \frac{1}{\sqrt{d}} \omega^{-jj'-ks_j} |j\rangle\langle j'| \tag{65}$$

where $s_j := (d-j)(d+j-1)/2$.

Proceeding similarly to Supplementary Eq. (52), we write

$$\begin{aligned}
C_{XZ^k} &= \sum_r (H_k \otimes H_k^*)|\phi_{0,r}\rangle\langle\phi_{0,r}|(H_k \otimes H_k^*)^\dagger &(66)\\
&= \sum_r (H_k \otimes H_k^*\sigma_Z^r)|\phi_{0,0}\rangle\langle\phi_{0,0}|(H_k^\dagger \otimes (\sigma_Z^r)^\dagger H_k^T) &(67)\\
&= \sum_r (\mathbb{1} \otimes H_k^*\sigma_Z^r H_k^T)|\phi_{0,0}\rangle\langle\phi_{0,0}|(\mathbb{1} \otimes H_k^*(\sigma_Z^r)^\dagger H_k^T) &(68)\\
&= \sum_r (\mathbb{1} \otimes \sigma_X^{d-r}\sigma_Z^{kr})|\phi_{0,0}\rangle\langle\phi_{0,0}|(\mathbb{1} \otimes (\sigma_X^{d-r}\sigma_Z^{kr})^\dagger) &(69)\\
&= \sum_r |\phi_{d-r,kr}\rangle\langle\phi_{d-r,kr}|\,, &(70)
\end{aligned}$$

which is diagonal in the Bell basis. In Supplementary Eq. (69), we used

$$H_k^*\sigma_Z^r H_k^T = \omega^{-kr(r+1)/2}\sigma_X^{d-r}\sigma_Z^{kr}, \tag{71}$$

and noted that the phase factor $\omega^{-kr(r+1)/2}$ disappears when multiplied by its conjugate.

SUPPLEMENTARY NOTE 4: ANALYSIS OF B92 PROTOCOL

Here we elaborate on our analysis of the B92 protocol. Recall that Alice sends one of two non-orthogonal states $\{|\phi_0\rangle, |\phi_1\rangle\}$ to Bob, and Bob randomly measures either in basis $B_0 = \{|\phi_0\rangle, |\overline{\phi_0}\rangle\}$ or basis $B_1 = \{|\phi_1\rangle, |\overline{\phi_1}\rangle\}$, where $\langle\phi_0|\overline{\phi_0}\rangle = \langle\phi_1|\overline{\phi_1}\rangle = 0$. They post-select on rounds where Bob gets outcome $|\overline{\phi_0}\rangle$ or $|\overline{\phi_1}\rangle$.

Since this is a prepare-and-measure protocol, we use the source-replacement scheme as outlined in the Results section. That is, Alice and Bob obtain constraints on the state ρ_{AB} in Eq. (13). The optimization problem is then defined as follows

$$\begin{aligned}
\text{Key-map POVM: } & Z_A = \{|0\rangle\langle 0|, |1\rangle\langle 1|\} &(72)\\
\text{Constraints: } & \langle \mathbb{1} \rangle = 1 &(73)\\
& \langle \Gamma_1 \rangle = p/2 &(74)\\
& \langle \Gamma_2 \rangle = p/2 + (1-p)\sin^2(\theta/2) &(75)\\
& \langle \sigma_X \otimes \mathbb{1} \rangle = \cos(\theta/2) &(76)\\
\text{Post-selection: } & G = \mathbb{1}_A \otimes \left[\frac{1}{2}\left(|\overline{\phi_0}\rangle\langle\overline{\phi_0}| + |\overline{\phi_1}\rangle\langle\overline{\phi_1}|\right)\right]^{1/2}. &(77)
\end{aligned}$$

Here, p is the depolarizing probability, $\sigma_X = |0\rangle\langle 1| + |1\rangle\langle 0|$, and the error and success operators are respectively

$$\begin{aligned}
\Gamma_1 &:= |0\rangle\langle 0| \otimes |\overline{\phi_0}\rangle\langle\overline{\phi_0}| + |1\rangle\langle 1| \otimes |\overline{\phi_1}\rangle\langle\overline{\phi_1}| &(78)\\
\Gamma_2 &:= |0\rangle\langle 0| \otimes |\overline{\phi_1}\rangle\langle\overline{\phi_1}| + |1\rangle\langle 1| \otimes |\overline{\phi_0}\rangle\langle\overline{\phi_0}|\,. &(79)
\end{aligned}$$

Note that the constraint in Supplementary Eq. (76) serves to constrain ρ_A and is of the form of Eq. (15) (see discussion around Eq. (14)). In principle one can add additional constraints on ρ_A, although we found this did not affect the key rate. In addition to the key map and constraints, note that we also needed to define the filter associated with Bob's

post-selection; see Eq. (18) in the Results section. Here we wrote the post-selection map in Kraus form: $\mathcal{G}(O) = GOG^\dagger$ for any operator O.

Supplementary Eqs. (72)-(77) define the optimization problem, and our results are shown in Fig. 6. We remark that our formulation of the dual problem has only 4 parameters, whereas the primal problem has 12 parameters, making the latter somewhat more difficult to solve.

SUPPLEMENTARY NOTE 5: ARBITRARY KEY-MAP POVMS

The Methods section proves our main result for the case where the key-map POVM is a projective measurement. Here we generalize this proof to arbitrary measurements.

First we rewrite the primal problem in terms of a coherence-like quantity, similar to what we did in the Methods section. Consider some POVM $P = \{P_j\}$ with $P_j \geqslant 0$ for each j, and $\sum_j P_j = \mathbb{1}$. Let us first define a generalized notion of coherence as follows,

$$\Phi_G(\rho, P) := D\left(\rho \middle\| \sum_j P_j \rho P_j\right). \tag{80}$$

We note that the second argument $\sum_j P_j \rho P_j$ is not necessarily normalized, although in general we have $\mathrm{Tr}(\sum_j P_j \rho P_j) \leqslant 1$, which follows from $P_j^2 \leqslant P_j$. In turn, this implies that Φ_G is non-negative:

$$\Phi_G(\rho, P) \geqslant 0\,. \tag{81}$$

Suppose Alice's measurement is an arbitrary POVM, $Z_A = \{Z_A^j\}$. Then, from Lemma 4 of Ref. [6], we have:

$$H(Z_A|E) \geqslant \Phi_G(\rho_{AB}, Z_A) \tag{82}$$

where E can be taken to purify ρ_{AB}. Hence we can define (or lower bound) the primal problem as

$$\alpha := \min_{\rho_{AB} \in \mathcal{C}} \Phi_G(\rho_{AB}, Z_A)\,, \tag{83}$$

which is analogous to Eq. (43).

One can then transform to the dual problem, as described in the Methods section. The only subtlety is that the analog of Eq. (50) can be written as an inequality:

$$\widehat{\Phi}_G(\rho_{AB}, Z_A) = \widehat{D}\big(\rho_{AB} || \mathcal{Z}_A(\rho_{AB})\big) \tag{84}$$

$$\geqslant \min_{\sigma_{AB} \in \mathcal{D}} \widehat{D}\big(\rho_{AB} || \mathcal{Z}_A(\sigma_{AB})\big). \tag{85}$$

The rest of the derivation proceeds as described in the Methods section.

SUPPLEMENTARY NOTE 6: STRONG DUALITY

In general the dual problem gives a lower bound on the primal problem, a fact called weak duality [7]. In the notation used in the Methods section, this means that

$$\widehat{\beta} \leqslant \widehat{\alpha}\,. \tag{86}$$

However, under certain conditions, the dual and primal problems are equivalent, which is called strong duality. Here we show that strong duality holds for our problem. That is, we prove Eq. (48), $\widehat{\beta} = \widehat{\alpha}$.

Slater's condition for a convex optimization problem is a sufficient criterion that guarantees strong duality [7]. For a problem with affine constraints, Slater's condition is satisfied when there is an element of the relative interior of the domain of optimization that satisfies the constraints. Thus, one way to guarantee strong duality for our problem is to show that there is a ρ_{AB} satisfying the constraints in Eq. (5) such that $\rho_{AB} > 0$. However, it is easy to imagine examples where Alice's and Bob's constraints specify a 0 eigenvalue for ρ_{AB}. For such examples, one would not satisfy Slater's condition, since there would be no $\rho_{AB} > 0$ consistent with the constraints.

We get around this issue as follows. We show that by slightly perturbing the constraints in a physical way, we can arrive at a "perturbed" problem which *does* satisfy Slater's condition and thus strong duality. Intuitively, changing the constraints infinitesimally should not change the solution to the primal problem by more than an infinitesimal amount; we prove this formally using the continuity of the coherence (see Supplementary Lemma 5 below). Thus solving the dual optimization (i.e., solving for $\widehat{\beta}$) can be done for slightly perturbed constraints, giving a solution arbitrarily close, and hence equivalent for all practical purposes, to the solution of the primal problem, $\widehat{\alpha}$. It is in this sense that we have strong duality in Eq. (48).

(For simplicity, we drop the subscript AB on ρ_{AB} for the remainder of this section, and just write ρ. Also, we assume that the $\{Z_A^j\}$ are projectors, and we replace $Z_A = \{Z_A^j\}$ with a generic set of projectors $\Pi = \{\Pi^j\}$.)

More precisely, we consider the following two optimization problems

$$\text{Problem 1: } a_1 = \min_{\rho \in \mathcal{S}_1} \Phi(\rho, \Pi) \tag{87}$$

$$\text{Problem 2: } a_2(\varepsilon) = \min_{\rho \in \mathcal{S}_2(\varepsilon)} \Phi(\rho, \Pi) \tag{88}$$

where one should recall that the primal problem objective function was identified in Eq. (43) as the coherence Φ. Here,

$$\mathcal{S}_1 := \{\rho \in \mathcal{H}_d : \rho \geqslant 0, \mathrm{Tr}(\rho\vec{\Gamma}) = \vec{\gamma}\}$$
$$\mathcal{S}_2(\varepsilon) := \{\rho \in \mathcal{H}_d : \rho > 0, \mathrm{Tr}(\rho\vec{\Gamma}) = (1 - d\varepsilon)\vec{\gamma} + \varepsilon \mathrm{Tr}(\vec{\Gamma})\}.$$

We call Supplementary Eq. (87) the unperturbed problem and Supplementary Eq. (88) the perturbed problem. In Supplementary Propositions 4 and 8, we shall prove that strong duality holds for the perturbed problem and that

$$\lim_{\varepsilon \to 0^+} a_2(\varepsilon) = a_1. \tag{89}$$

By choosing ε sufficiently small, solving the dual perturbed problem will then yield a result that is arbitrarily close to the solution to the primal problem.

Supplementary Proposition 4: The strong duality property holds for the perturbed problem.

Proof. We prove this by showing that Slater's condition is satisfied for the perturbed primal problem, which implies strong duality for the perturbed problem [7]. Slater's condition for this convex optimization problem with affine constraints is satisfied when there is a $\rho > 0$ that satisfies the constraints.

We begin by considering a map from the domain $\mathcal{S}_1$ to $\mathcal{S}_2(\varepsilon)$. Let the map $\mathcal{M}_\varepsilon$ act on a state ρ via

$$\mathcal{M}_\varepsilon(\rho) = (1 - d\varepsilon)\rho + \varepsilon \mathbb{1}. \tag{90}$$

Note that if $\rho \in \mathcal{S}_1$, then $\mathcal{M}_\varepsilon(\rho) \in \mathcal{S}_2(\varepsilon)$.

We take the given set of constraints to be physical, i.e., $\mathcal{S}_1$ is non-empty. By the map $\mathcal{M}_\varepsilon$, $\mathcal{S}_2(\varepsilon)$ is non-empty as well. Then, as $\rho > 0$ for all $\rho \in \mathcal{S}_2(\varepsilon)$, Slater's condition is satisfied. □

Before proving the continuity of the perturbation, we note that coherence Φ has some nice properties. One property that we will make explicit use of is its *continuity* in the state ρ, which we prove in the following lemma. After completion of this work, Ref. [8] proved the same lemma in their article.

Supplementary Lemma 5: Let ρ and σ be two density operators on a Hilbert space of dimension d. Suppose they are close in trace distance $T(\tau, \tau') := (1/2)\mathrm{Tr}|\tau - \tau'|$, in particular, suppose $T(\rho, \sigma) \leqslant 1/e$. Then the coherences of ρ and σ are nearly equal:

$$\begin{aligned} \Delta\Phi &:= |\Phi(\rho, \Pi) - \Phi(\sigma, \Pi)| \\ &\leqslant 2[T(\rho, \sigma)\log_2 d - T(\rho, \sigma)\log_2 T(\rho, \sigma)]. \end{aligned} \tag{91}$$

Proof. The proof uses Fannes' inequality, which states that

$$|H(\rho) - H(\sigma)| \leqslant T(\rho, \sigma)\log_2 d - T(\rho, \sigma)\log_2 T(\rho, \sigma), \tag{92}$$

which holds so long as $T(\rho, \sigma) \leqslant 1/e$. Note that, because of the monotonicity of the trace distance under quantum channels, we also have $T(\rho_\Pi, \sigma_\Pi) \leqslant 1/e$, where $\rho_\Pi = \sum_j \Pi^j \rho \Pi^j$ and $\sigma_\Pi = \sum_j \Pi^j \sigma \Pi^j$. Hence Supplementary Eq. (92) also holds for the states ρ_Π and σ_Π.

Noting that $\log \rho_\Pi = \sum_j \Pi^j (\log \rho_\Pi) \Pi^j$, we have

$$H(\rho_\Pi) = -\mathrm{Tr}(\rho \log \rho_\Pi),$$

and hence we can rewrite the coherence as

$$\Phi(\rho, \Pi) = H(\rho_\Pi) - H(\rho).$$

This allows us to bound the coherence difference:

$$\begin{aligned}
\Delta\Phi &= |H(\rho_\Pi) - H(\sigma_\Pi) + H(\sigma) - H(\rho)| && (93)\\
&\leqslant |H(\rho_\Pi) - H(\sigma_\Pi)| + |H(\sigma) - H(\rho)| && (94)\\
&\leqslant T(\rho_\Pi, \sigma_\Pi) \log_2 d - T(\rho_\Pi, \sigma_\Pi) \log_2 T(\rho_\Pi, \sigma_\Pi) + T(\rho, \sigma) \log_2 d - T(\rho, \sigma) \log_2 T(\rho, \sigma) && (95)\\
&\leqslant 2[T(\rho, \sigma) \log_2 d - T(\rho, \sigma) \log_2 T(\rho, \sigma)], && (96)
\end{aligned}$$

where the last line uses $T(\rho_\Pi, \sigma_\Pi) \leqslant T(\rho, \sigma)$, as well as the monotonicity of $(-x \log x)$ over the interval $x \in [0, 1/e]$. □

To prove Supplementary Eq. (89), we first state the following two technical lemmas.

Supplementary Lemma 6: There exists a $\rho \in \mathcal{S}_2(\varepsilon)$ such that $\Phi(\rho, \Pi)$ is within $\mathcal{O}(\varepsilon)$ of the unperturbed solution, a_1.

Proof. Let $\overline{\rho} \in \mathcal{S}_1$ be a positive semidefinite matrix that minimizes the unperturbed problem, i.e.,

$$a_1 = \Phi(\overline{\rho}, \Pi)\,. \tag{97}$$

Consider the corresponding positive definite matrix $\mathcal{M}_\varepsilon(\overline{\rho}) \in \mathcal{S}_2(\varepsilon)$, where $\mathcal{M}_\varepsilon : \mathcal{S}_1 \to \mathcal{S}_2(\varepsilon)$ is the injection defined in Supplementary Eq. (90). We argue that this injection does not change the objective function much, due to continuity of coherence. Note that the states $\overline{\rho}$ and $\mathcal{M}_\varepsilon(\overline{\rho})$ are close in trace distance:

$$T(\overline{\rho}, \mathcal{M}_\varepsilon(\overline{\rho})) = d\varepsilon T(\overline{\rho}, \mathbb{1}/d) \leqslant d\varepsilon\,. \tag{98}$$

From Supplementary Eq. (91) we have that

$$|\Phi(\overline{\rho}, \Pi) - \Phi(\mathcal{M}_\varepsilon(\overline{\rho}), \Pi)| \leqslant -2d\varepsilon \log_2 \varepsilon\,, \tag{99}$$

which proves the statement. □

Supplementary Lemma 7: There exists a $\rho \in \mathcal{S}_1$ such that $\Phi(\rho, \Pi)$ is within $\mathcal{O}(\varepsilon)$ of the perturbed solution, $a_2(\varepsilon)$.

Proof. As in Supplementary Note 2, we consider the trace constraints rephrased (via the Gram-Schmidt process) in terms of an orthonormal set of operators $\{\Delta_i\}$, i.e.,

$$\mathrm{Tr}(\rho \vec{\Gamma}) = \vec{\gamma} \quad \to \quad \mathrm{Tr}(\rho \vec{\Delta}) = \vec{\delta}\,, \tag{100}$$

and we extend the set to an orthonormal basis of Hermitian operators:

$$\{\Delta_i\} \cup \{\Xi_j\}\,. \tag{101}$$

For $\rho_1 \in \mathcal{S}_1$ and $\rho_2 \in \mathcal{S}_2(\varepsilon)$, the most general expressions for these states are:

$$\begin{aligned}
\rho_1 &= \vec{\delta} \cdot \vec{\Delta} + \vec{\xi}_1 \cdot \vec{\Xi} && (102)\\
\rho_2 &= (\vec{\delta} + \vec{p}) \cdot \vec{\Delta} + \vec{\xi}_2 \cdot \vec{\Xi}, && (103)
\end{aligned}$$

where $\vec{\xi}_1$ is any vector that enforces the positive semidefiniteness of ρ_1 and $\vec{\xi}_2$ is any vector that enforces the positive definiteness of ρ_2. In Supplementary Eq. (103), we used the fact that the constraint perturbation from the unperturbed problem in Supplementary Eq. (87) to the perturbed problem in Supplementary Eq. (88) takes the form $\vec{\delta} \to \vec{\delta} + \vec{p}$, with $\vec{p} := \varepsilon \vec{p}_0$, and

$$\vec{p}_0 := \mathrm{Tr}(\vec{\Delta}) - d\,\vec{\delta}. \tag{104}$$

In what follows, we study the geometry of the set of possible $\vec{\xi}_1$ and $\vec{\xi}_2$, thereby finding the most general form for ρ_1 and ρ_2.

For all states $|i\rangle$ in the Hilbert space $\mathcal{H}$, the positive (semi)definiteness requirements are equivalent to the following statements, for all $|i\rangle$

$$\langle i|\rho_1|i\rangle = \vec{\delta} \cdot \langle i|\vec{\Delta}|i\rangle + \vec{\xi}_1 \cdot \langle i|\vec{\Xi}|i\rangle \geq 0 \tag{105}$$

$$\langle i|\rho_2|i\rangle = (\vec{\delta} + \vec{p}) \cdot \langle i|\vec{\Delta}|i\rangle + \vec{\xi}_2 \cdot \langle i|\vec{\Xi}|i\rangle > 0. \tag{106}$$

Define the infinite-dimensional operators C and D with $(C)_{ij} := \langle i|(\vec{\Xi})_j|i\rangle$ and $(D)_{ij} := \langle i|(\vec{\Delta})_j|i\rangle$. Then the $\vec{\xi}$ which enforce the positive (semi)definiteness requirements are those which satisfy the following infinite vector of inequalities

$$C \cdot \vec{\xi}_1 \geq -D \cdot \vec{\delta} = \vec{s} \tag{107}$$

$$C \cdot \vec{\xi}_2 > -D \cdot \vec{\delta} - \varepsilon D \cdot \vec{p}_0 = \vec{s} + \varepsilon \vec{t}, \tag{108}$$

where the right sides are fixed quantities, so we defined $\vec{s} := -D \cdot \vec{\delta}$ and $\vec{t} := -D \cdot \vec{p}_0$.

For each $|i\rangle$, the solution space of $\vec{\xi}_1$ and $\vec{\xi}_2$ satisfying the inequality constraint is a hyperplane defined by

$$C_{i1}\left(\vec{\xi}_1\right)_1 + C_{i2}\left(\vec{\xi}_1\right)_2 + \cdots \geq s_i \tag{109}$$

$$C_{i1}\left(\vec{\xi}_2\right)_1 + C_{i2}\left(\vec{\xi}_2\right)_2 + \cdots > s_i + \varepsilon t_i. \tag{110}$$

The regions of all possible $\vec{\xi}_1$ and $\vec{\xi}_2$ are the intersections of all of the constrained regions, each bounded by a hyperplane. Since $\mathcal{S}_1$ and $\mathcal{S}_2(\varepsilon)$ are non-empty, the solution sets for $\vec{\xi}_1$ and $\vec{\xi}_2$ are non-empty. Perturbing the constraints is equivalent to shifting each hyperplane slightly (by εt_i for the ith hyperplane). For sufficiently small ε, each $\vec{\xi}_2$ in the perturbed solution set is within $\mathcal{O}(\varepsilon)$ in Euclidean distance of a $\vec{\xi}_1$ in the unperturbed solution set [9].

Now, let $\hat{\rho}_2 \in \mathcal{S}_2(\varepsilon)$ be a positive definite matrix that minimizes the perturbed problem, i.e.,

$$a_2(\varepsilon) = \Phi(\hat{\rho}_2, \Pi). \tag{111}$$

In terms of the basis in Supplementary Eq. (101),

$$\hat{\rho}_2 = (\vec{\delta} + \varepsilon \vec{p}_0) \cdot \vec{\Delta} + \hat{\vec{\xi}}_2 \cdot \vec{\Xi}, \tag{112}$$

for a vector $\hat{\vec{\xi}}_2$ that enforces positive definiteness for $\hat{\rho}_2$.

By the previous discussion, there exist a $\hat{\vec{\xi}}_1$ which enforces positive semidefiniteness for a matrix satisfying the unperturbed trace constraints that is within $\mathcal{O}(\varepsilon)$ of $\hat{\vec{\xi}}_2$. That is, the vector

$$\vec{v} := \hat{\vec{\xi}}_2 - \hat{\vec{\xi}}_1 \tag{113}$$

has a norm $|\vec{v}|$ that is $\mathcal{O}(\varepsilon)$, and hence each component of $\vec{v}$ is no larger in magnitude than $\mathcal{O}(\varepsilon)$. We define

$$\hat{\rho}_1 := \vec{\delta} \cdot \vec{\Delta} + \hat{\vec{\xi}}_1 \cdot \vec{\Xi} \in \mathcal{S}_1. \tag{114}$$

The difference between Supplementary Eqs. (112) and (114)

$$\hat{\rho}_2 - \hat{\rho}_1 = \varepsilon \vec{p}_0 \cdot \vec{\Delta} + \vec{v} \cdot \vec{\Xi} \tag{115}$$

has trace norm $\mathcal{O}(\varepsilon)$, as it is a sum of Hermitian operators with coefficients that are $\mathcal{O}(\varepsilon)$. Hence we have constructed an element $\hat{\rho}_1 \in \mathcal{S}_1$ that is within $\mathcal{O}(\varepsilon)$ in trace distance of $\hat{\rho}_2$. Then by continuity of coherence in Supplementary Lemma 5, the objective function value $\Phi(\hat{\rho}_1, \Pi)$ is within $\mathcal{O}(\varepsilon)$ of $a_2(\varepsilon)$. □

Supplementary Proposition 8: For $a_1, a_2(\varepsilon)$ defined in Supplementary Eqs. (87) and (88),

$$\lim_{\varepsilon \to 0^+} a_2(\varepsilon) = a_1. \tag{116}$$

Proof. Supplementary Lemma 6 implies that

$$\lim_{\varepsilon \to 0^+} a_2(\varepsilon) \leqslant a_1 \,. \tag{117}$$

Supplementary Lemma 7 implies that

$$\lim_{\varepsilon \to 0^+} a_2(\varepsilon) \geqslant a_1 \,. \tag{118}$$

Combining these two facts gives the desired result. □

SUPPLEMENTARY REFERENCES

[1] Konig, R., Renner, R. & Schaffner, C. The operational meaning of min-and max-entropy. *IEEE Transactions on Information Theory* **55**, 4337–4347 (2009).

[2] Lo, H.-K., Chau, H. F. & Ardehali, M. Efficient quantum key distribution scheme and a proof of its unconditional security. *J. Cryptology* **18**, 133–165 (2004).

[3] Lo, H.-K., Curty, M. & Qi, B. Measurement-Device-Independent Quantum Key Distribution. *Physical Review Letters* **108**, 130503 (2012).

[4] Bandyopadhyay, S., Boykin, P. O., Roychowdhury, V. & Vatan, F. A new proof for the existence of mutually unbiased bases. *Algorithmica* **34**, 512–528 (2001).

[5] Bennett, C. H. Quantum cryptography using any two nonorthogonal states. *Physical Review Letters* **68**, 3121–3124 (1992).

[6] Coles, P. J., Yu, L., Gheorghiu, V. & Griffiths, R. B. Information-theoretic treatment of tripartite systems and quantum channels. *Physical Review A* **83**, 062338 (2011).

[7] Boyd, S. & Vandenberghe, L. *Convex Optimization* (Cambridge University Press, 2004).

[8] Winter, A. & Yang, D. Operational resource theory of coherence 1–10 (2015). arXiv:1506.07975v2.

[9] Hu, H. & Wang, Q. On approximate solutions of infinite systems of linear inequalities. *Linear Algebra and its Applications* **114**, 429–438 (1989).
